# First Days in the Life of Naive Human B Lymphocytes Infected with Epstein-Barr Virus

**DOI:** 10.1128/mBio.01723-19

**Published:** 2019-09-17

**Authors:** Dagmar Pich, Paulina Mrozek-Gorska, Mickaël Bouvet, Atsuko Sugimoto, Ezgi Akidil, Adam Grundhoff, Stephan Hamperl, Paul D. Ling, Wolfgang Hammerschmidt

**Affiliations:** a Research Unit Gene Vectors, Helmholtz Zentrum München, German Research Center for Environmental Health and German Center for Infection Research (DZIF), Munich, Germany; b Heinrich Pette Institute, Leibniz Institute for Experimental Virology, Hamburg, Germany; c Institute of Epigenetics and Stem Cells, Helmholtz Zentrum München, German Research Center for Environmental Health, Munich, Germany; d Department of Molecular Virology and Microbiology, Baylor College of Medicine, Houston, Texas, USA; Johns Hopkins University; Johns Hopkins Bloomberg School of Public Health

**Keywords:** B lymphocytes, human herpesviruses, reprogramming, transformation

## Abstract

Epstein-Barr virus (EBV) infects and activates resting human B lymphocytes, reprograms them, induces their proliferation, and establishes a latent infection in them. In established EBV-infected cell lines, many viral latent genes are expressed. Their roles in supporting the continuous proliferation of EBV-infected B cells *in vitro* are known, but their functions in the early, prelatent phase of infection have not been investigated systematically. In studies during the first 8 days of infection using derivatives of EBV with mutations in single genes of EBVs, we found only Epstein-Barr nuclear antigen 2 (EBNA2) to be essential for activating naive human B lymphocytes, inducing their growth in cell volume, driving them into rapid cell divisions, and preventing cell death in a subset of infected cells. EBNA-LP, latent membrane protein 2A (LMP2A), and the viral microRNAs have supportive, auxiliary functions, but mutants of LMP1, EBNA3A, EBNA3C, and the noncoding Epstein-Barr virus with small RNA (EBERs) had no discernible phenotype compared with wild-type EBV. B cells infected with a double mutant of EBNA3A and 3C had an unexpected proliferative advantage and did not regulate the DNA damage response (DDR) of the infected host cell in the prelatent phase. Even EBNA1, which has very critical long-term functions in maintaining and replicating the viral genomic DNA in established cell lines, was dispensable for the early activation of infected cells. Our findings document that the virus dose is a decisive parameter and indicate that EBNA2 governs the infected cells initially and implements a strictly controlled temporal program independent of other viral latent genes. It thus appears that EBNA2 is sufficient to control all requirements for clonal cellular expansion and to reprogram human B lymphocytes from energetically quiescent to activated cells.

## Abstract (precis)

The preferred target of Epstein-Barr virus (EBV) is human resting B lymphocytes. We found that their infection induces a well-coordinated, time-driven program that starts with a substantial increase in cell volume, followed by cellular DNA synthesis after 3 days and subsequent rapid rounds of cell divisions on the next day accompanied by some DNA replication stress (DRS). Two to 3 days later, the cells decelerate and turn into stably proliferating lymphoblast cell lines. With the aid of 16 different recombinant EBV strains, we investigated the individual contributions of EBV’s multiple latent genes during early B-cell infection and found that many do not exert a detectable phenotype or contribute little to EBV’s prelatent phase. The exception is EBNA2 that is essential in governing all aspects of B-cell reprogramming. EBV relies on EBNA2 to turn the infected B lymphocytes into proliferating lymphoblasts preparing the infected host cell for the ensuing stable, latent phase of viral infection. In the early steps of B-cell reprogramming, viral latent genes other than EBNA2 are dispensable, but some, EBNA-LP, for example, support the viral program and presumably stabilize the infected cells once viral latency is established.

## INTRODUCTION

In 1964, Epstein, Achong, and Barr identified a new herpesvirus in a cell line derived from Burkitt’s lymphoma (1), and 3 years later, two groups independently found that this virus, now termed Epstein-Barr virus (EBV), can transform human primary B lymphocytes into lymphoblastoid cell lines (LCLs) that proliferate in culture (2–4).

Initially, many groups have focused on the identification of the viral genes and factors expressed in the latently infected and proliferating human B cells, i.e., EBV’s latent genes. This explorative period was followed by a second wave of publications starting in 1989 (5, 6), which studied the genetic requirements of the identified latent viral genes in this B-cell model. We found that Epstein-Barr nuclear antigen 2 (EBNA2) was an essential viral gene (7), and other early publications suggested that the latent membrane protein 1 (LMP1), Epstein-Barr nuclear antigen 3A (EBNA3A), and EBNA3C were essential for the generation of LCLs from *in vitro*-infected human B lymphocytes, whereas LMP2A, LMP2B, EBNA3B, and the two noncoding RNAs, EBER1 and -2, were dispensable but contributed to this process (8). With the emergence of more sophisticated genetic approaches, it was possible to study EBV’s latent genes more accurately. The synthetic assembly of mini-EBV genomes (9–11) and the eventual cloning of the entire EBV genome in Escherichia coli (12) opened the field for the unlimited genetic analysis of all viral genes (and EBV’s *cis*-acting elements) (13). From these studies (and in the context of an otherwise genetically unaltered EBV genome), it became clear that LMP1 (14) and EBNA3A (9, 15) are dispensable, and only EBNA2 is essential to induce and maintain LCLs from *in vitro* EBV-infected B lymphocytes (16). EBNA3C is required to maintain the proliferation of LCLs long term (17, 18) but is dispensable in rare *p16^INK4a^*-deficient B cells (19).

Very much in contrast to many viruses, EBV does not start the *de novo* synthesis of virus progeny upon infection; rather, it initiates a latent infection (20, 21). It was therefore surprising to learn that certain lytic viral genes are expressed in newly infected B cells (20, 22–24). Their expression is transient, only, but some are essential for the emergence of lymphoblastoid cell lines (16), suggesting that EBV uses a set of viral genes in the first few days of infection that differs from the set of viral genes expressed in established, stable, and latently infected lymphoblastoid cell lines (25).

Here, we sought to analyze the individual genetic contributions of all latent viral genes to the activation and transformation of human B lymphocytes during the first 8 days following infection. Our experiments suggest that the reprogramming of newly infected cells, which follows a very strict, time-controlled scheme is orchestrated solely by EBNA2, a viral factor that has been shown to be essential for maintaining B-cell transformation previously (7).

## RESULTS

### The virus dose is an important parameter of cell survival and proliferation in the prelatent phase of EBV infection.

We started this study by refining our previous data (26) to determine the parameters of infection and to identify the optimal ratio of EBV versus human primary B lymphocytes. We infected them with different multiplicities of infection (MOIs) of wt/B95.8 (2089) EBV (Table 1) and counted the absolute number of viable lymphocytes and emerging B blasts by fluorescence-activated cell sorting (FACS) employing calibrated allophycocyanin (APC) beads (Calibrite; Becton, Dickinson) as a volume standard. In this analysis, we also investigated the binding of annexin V, an early indicator of apoptosis. Concomitantly, we determined the fraction of EBNA2-positive B cells. Towards this end, we fixed and permeabilized the cells, stained them with an anti-EBNA2 antibody coupled with Alexa 647, and analyzed the stained cells by flow cytometry.

**TABLE 1:** Features of the EBV strains used in infection experiments

EBV strain	Description or genotype^*a*^	Specification	Genealogy	Reference or source
wt/B95.8 (2089)	Wild type, 13 miRNAs, *hpt*	Reference EBV strain		12
wt/B95.8 (6001)	Wild type, 13 miRNAs, *pac*	Reference EBV strain	Based on wt/B95.8 (2089)	This study
r_wt/B95.8 (6008)	Wild type, 44 miRNAs, *pac*	Deletion in wt/B95.8 (2089) restored	Based on wt/B95.8 (2089)	This study
wt/B95.8 (5750)	Wild type, 13 miRNAs, *hpt*	Six BamHI-W repeats	Based on wt/B95.8 (2089)	This study
ΔEBNA1 (6285) mutant	Knockout of EBNA1, *pac*	AUG-to-TAG mutation of EBNA1’s translational start codon	Based on r_wt/B95.8 (6008)	This study
ΔEBNA-LP (5969) mutant	EBNA-LP knockout, *pac*	Six BamHI-W repeats, in-frame translational stop codon in each W2 exon of EBNA-LP	Based on wt/B95.8 (2089)	This study
ΔEBNA2 (5968) mutant	EBNA2 knockout), *pac*	AUG-to-TGA mutation of EBNA2’s translational start codon	Based on wt/B95.8 (2089)	This study
ΔEBNA3A (6077) mutant	Knockout of EBNA3A, *pac*	Insertional mutagenesis of EBNA3A	Based on wt/B95.8 (6001)	This study
ΔEBNA3C (6123) mutant	Knockout of EBNA3C, *pac*	Two in-frame stop codons in exons 1 and 2 of EBNA3C	Based on wt/B95.8 (6001)	This study
ΔEBNA3A/C (6331) mutant	Knockout of EBNA3A and 3C, *pac*	Insertional mutagenesis of EBNA3A	Based on ΔEBNA3C (6123) mutant	This study
ΔLMP1 (2597) mutant	LMP1 knockout, *hpt*	Deletion of *BNLF1*	Based on wt/B95.8 (2089)	14
ΔLMP2A (2525) mutant	LMP2 knockout, *hpt*	Cre-mediated deletion of first exon of LMP2A	Based on wt/B95.8 (2089)	97
ΔmiR (4027) mutant	No viral miRNAs, *hpt*	Scrambled pre-miRNA loci	Based on wt/B95.8 (2089)	42
r_ ΔmiR (6338) mutant	No viral miRNAs, *pac*	Scrambled pre-miRNA loci	Based on r_wt/B95.8 (6008)	This study
ΔEBER (6431) mutant	Knockout of EBER1 and -2, *pac*	Insertional mutagenesis	Based on r_wt/B95.8 (6008)	This study
ΔEBER/ΔmiR (6432) mutant	Knockout of EBER1 and -2, miRNAs, *pac*	Scrambled pre-miRNA loci, insertional mutagenesis	Based on r_ ΔmiR (6338) mutant	This study

a *hpt*, hygromycin B phosphotransferase gene; encodes resistance to hygromycin B; *pac*, puromycin *N*-acetyltransferase; encodes resistance to puromycin.

**FIG 1:**
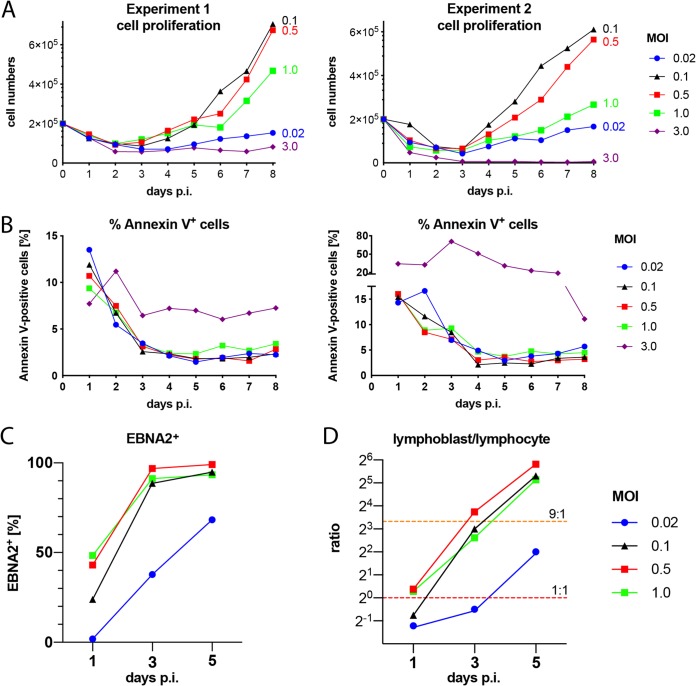
Evaluation of parameters and conditions supporting EBV infection of naive B lymphocytes. (A) Primary naive B lymphocytes were sorted and infected with wt/B95.8 (2089) EBV using the indicated multiplicities of infection (MOI). The number of proliferating, growth-transformed B cells was recorded by flow cytometry daily, as described in Materials and Methods and in Fig. 2A of our recent publication (26). Two experiments with B cells from two different donors are shown. (B) Annexin V binding of infected B lymphocytes from the two donors analyzed in panel A is provided. (C) The fraction of EBNA2-positive cells in B cells on days 1, 3, and 5 p.i. is shown as a function of MOI. (D) The ratio of lymphoblasts versus lymphocytes, as determined by forward- and side-scatter flow cytometry analysis was calculated with B cells infected with wt/B95.8 (2089) and different MOIs as indicated on days 1, 3, and 5 p.i. The horizontal dashed red line indicates a 1:1 ratio, and the orange line indicates a 9:1 ratio of lymphoblasts versus lymphocytes. Panels A and B show the results from two representative experiments out of three, and panels C and D show the results from one representative experiment out of three.

**FIG 2:**
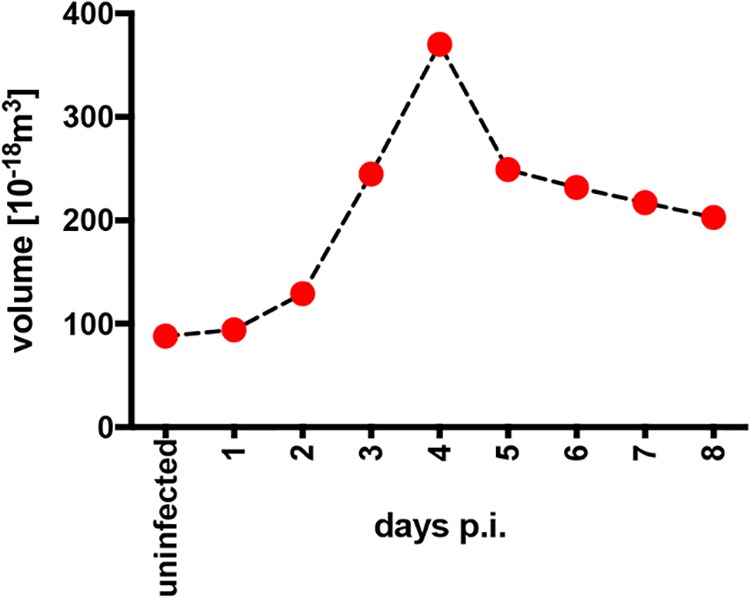
Cell volume of naive B lymphocytes infected with EBV. FACS-sorted naive B lymphocytes from adenoid tissue were left uninfected or were infected with wt/B95.8 (2089) EBV with an MOI of 0.1 and cultivated for the indicated days. After Ficoll gradient centrifugation, microscopic images of the samples were recorded with a Tali image-based cytometer (Thermo Fisher) and analyzed for their cellularity and the cells’ diameters, which were determined with the aid of calibration beads, as described in Materials and Methods. Based on the cell diameter, the mean volume of at least 200 cells per time point was calculated, assuming perfect spheres.

The concentration of infectious particles in stocks of EBV is measured by infecting Raji cells and determining the fraction of green fluorescent protein (GFP)-positive cells by flow cytometry 3 days later, as described in detail in Fig. 2 of our previous work (26). In this paper, we also noticed that Daudi cells are much more permissive for EBV infection than Raji cells, documenting the fact that we underestimate the virus concentration by at least a factor of 10 or more when using Raji cells for virus quantification (Fig. 2B in reference 26). In two independent experiments with sorted naive B lymphocytes from adenoids, we confirmed that an MOI of 0.1 based on green Raji units (GRUs) was optimal (Fig. 1A), because this dose reproducibly yielded the highest numbers of B cells 8 days postinfection (p.i.). Lower MOIs were inferior, but it was unexpected to learn that virus doses beyond 0.1 also resulted in lower cell numbers on day 8 p.i., although the fraction of EBNA2+ cells was initially higher than with cells infected with an MOI of 0.1 (Fig. 1C). Also, annexin V binding was dramatically increased when the cells were infected with a high MOI of 3.0 (Fig. 1B). We learned from these experiments that an MOI of 0.1 on Raji cells equates to an MOI of 1.0 or higher on B lymphocytes. Our results also indicated that EBV’s success is dose dependent and has a well-defined, but relatively narrow, virus-to-host cell ratio that optimally supports the early survival, activation, and reprogramming of EBV’s target B lymphocytes. Consequently, we chose an MOI of 0.1 GRU for most subsequent experiments.

### EBV reprograms naive human B lymphocytes in discrete steps.

We isolated primary human B lymphocytes from adenoid tissue and sorted the fraction of quiescent naive B cells (IgD^+^/IgH^+^, CD38^−^, CD27^−^) for our infection experiments. The naive B cells were infected with the recombinant EBV based on the B95.8 strain termed wt/B95.8 (2089) (12) (Table 1) using an MOI of 0.1. We recorded the diameter of the viable cells daily by microscopic imaging and calculated their volume assuming a spherical shape. Uninfected cells had a diameter of 5.5 ± 0.5 μm (mean and standard deviation), which gradually increased to close to 8.9 μm on day 4 postinfection (p.i.) but decreased to 7.5 ± 1.2 μm on day 8 p.i. (27). Similarly, the inferred volume of the cells increased from 87 × 10^−18^ m^3^ to 357 × 10^−18^ m^3^ on day 4 p.i. and declined to about 220 × 10^−18^ m^3^ (mean values) on day 8 p.i. (Fig. 2), indicating a roughly 4-fold change within this period of infection.

We repeated the experiments with sorted naive B lymphocytes that were labeled with an intracellular dye (cell trace violet [CTV]) prior to infection to monitor the cell doublings over time. Four different EBV strains were used for infection, which represent four versions of the wt/B95.8 strain (12) but differ in certain genotypic features (Table 1). Briefly, wt/B95.8 (2089) and wt/B95.8 (6001) are identical recombinant EBVs but contain *hpt* or *pac* in the virus producer cells, conferring resistance to hygromycin B and puromycin, respectively. Based on wt/B95.8 (2089), wt/B95.8 (5750) was engineered to contain six complete copies of the BamHI-W-repeat cluster (see below for details).

The fourth versions of the wt/B95.8 strain termed r_wt/B95.8 (6008) adds to our collection of available EBV strains (Table 1). The B95.8 reference strain of EBV originates from a lymphoblastoid cell line obtained by infecting marmoset monkey peripheral blood leukocytes with EBV from a patient with infectious mononucleosis (28, 29). The B95.8 EBV strain readily immortalizes human B lymphocytes and has been studied for decades because the size of its genome was recognized to be smaller than those of most other EBV field strains, an advantage in the early search for EBV’s immortalizing functions (30, 31). Its genome, however, suffers from a unique deletion (32, 33) that affects the cluster of EBV’s microRNAs (miRNAs) (34), several protein-encoding viral genes (35), and a second copy of EBV’s lytic origin of DNA replication (36). It has remained uncertain whether these viral functions might contribute to B-cell immortalization or establishment of latency. To address this issue, we engineered a derivative of wt/B95.8 (2089) termed r_wt/B95.8 (6008) EBV, in which the 12-kb deletion present in the B95.8 EBV strain was restored with the autologous sequences of the M-ABA EBV isolate (32) to represent the genetic content of common field strains of EBV (31, 37). The DNA sequence of the r_wt/B95.8 (6008) EBV strains corresponds to the reference EBV genomic sequences with accession numbers AJ507799 and NC_007605.

With the four related B95.8 EBV derivates wt/B95.8 (2089), wt/B95.8 (6001), wt/B95.8 (5750), and r_wt/B95.8 (6008), summarized in Table 1, we infected primary sorted naive B lymphocytes and analyzed them by flow cytometry daily to record cell doublings, annexin V binding, and numbers of intact cells for 8 days. Cellular DNA synthesis and cell cycle distribution were investigated after metabolic labeling with 5′-bromo-2′-deoxyuridine (BrdU) for 1 h and subsequent immunodetection and flow cytometry analysis of the cellular DNA content.

**FIG 3:**
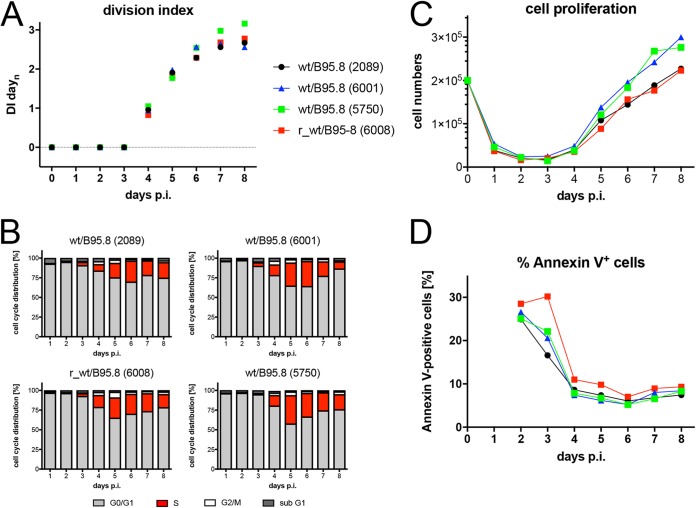
Activation kinetics of naive B lymphocytes infected with four different recombinant wild-type EBV strains. Sorted naive B lymphocytes isolated from adenoid tissue were loaded with an intracellular dye (cell trace violet [CTV]; Thermo Fisher Scientific). The cells were infected with the four indicated EBV strains with an MOI of 0.1. The strains are wild type with respect to EBV’s latent genes but have different genotypes, as specified in Table 1. (A) The kinetics of cell division of the infected cells were analyzed by flow cytometry, and the resulting division index (DI) was calculated and plotted. DI indicates the average number of cell divisions a cell in the starting populations has undergone, including the peak of undivided cells. (B) Cells were incubated with 5-bromo-2′-deoxyuridine (BrdU) for 1 h prior to harvest and analyzed by flow cytometry after staining with a BrdU specific antibody. The percentage of cells in the different phases of the cell cycle were calculated. (C) Cell numbers of viable cells were analyzed by flow cytometry as described in reference 26 and plotted. The initial cell numbers in this experiment were 2 × 10^5^ per well, as indicated. (D) Annexin V binding of infected cells was analyzed by flow cytometry. The results from one representative experiment out of three experiments with B lymphocytes from three individual donors are shown.

The naive B lymphocytes did not divide within the first 3 days of infection (Fig. 3A), indicating that the increase in cell volume (Fig. 2) reflects the metabolic growth of the infected and activated cells only. The first cell division occurred on day 4, followed by a short period of rapidly dividing cells until day 6 postinfection (p.i.). Thereafter, the dividing cells decelerated considerably to adopt a rate of cell divisions also seen in established lymphoblastoid cell lines. BrdU incorporation was first detected on day 3 p.i. but never earlier, followed by a rapid increase of the fraction of S-phase cells that reached a maximum on day 5 or 6 p.i. (Fig. 3B). Cell numbers dropped dramatically and, in most experiments, up to 70% of the initially infected cells were lost within the first 3 days of cell culture (Fig. 3C). Cell numbers increased from day 4 onwards in parallel with the onset of cell divisions (Fig. 3A). A high and often variable fraction of cells bound annexin V within the first 3 days of cell culture, but starting on day 4, generally more than 90% of the intact viable cells became annexin V negative (Fig. 3D). The phenotypes of the cells infected with the four individual wild-type EBV stocks did not differ much but merely reflected the experimental variability of the assay. The novel EBV strain r_wt/B95.8 (6008), which gets as close as possible to an EBV field strain while preserving the context of the prototypic B95.8 EBV, does not reveal a discrete phenotype in this set of experiments but opens new possibilities for investigating EBV-encoded miRNAs.

### Reprogramming of resting B lymphocytes from peripheral blood.

We asked if the phenotype of the EBV-infected naive B lymphocytes might reflect their origin from secondary lymphatic tissue. Therefore, we repeated the previous experiment with B cells isolated from peripheral blood, which contains resting, nonactivated naive and memory B lymphocytes only. Upon infection of peripheral B lymphocytes from two donors with wt/B95.8 (2089), we recorded cell size and granularity, cell numbers, division index, and rate of apoptosis by flow cytometry (see Fig. S1 in the supplemental material), but were unable to analyze cellular DNA synthesis due to low initial cell numbers. Similar to sorted naive B lymphocytes from adenoid tissue, the majority of peripheral B cells died within the first 3 days, but the surviving cells were rapidly activated (Fig. S1), started to proliferate on day 4 p.i., and reduced the rate of proliferation on day 6. In general, the phenotypic differences of infected naive B lymphocytes from adenoids versus B lymphocytes from peripheral blood were minor, indicating that B lymphocytes from different sources apparently followed the identical time scheme.

10.1128/mBio.01723-19.1 FIG S1 Activation kinetics of peripheral B lymphocytes isolated from PBMCs and infected with wt/B95.8 (2089) EBV. Resting B lymphocytes from PBMCs were infected with wt/B95.8 (2089) EBV with an MOI of 0.1 and analyzed for the division index, cell numbers, and percentage of annexin V-positive cells. Infection experiments with PBMCs from three different donors were performed; shown is one representative set of results. Download FIG S1, PDF file, 0.1 MB. Copyright © 2019 Pich et al. 2019 Pich et al. This content is distributed under the terms of the Creative Commons Attribution 4.0 International license.

### CD40 activation and IL-4 induce a comparable program of B-cell activation and proliferation.

**FIG 4:**
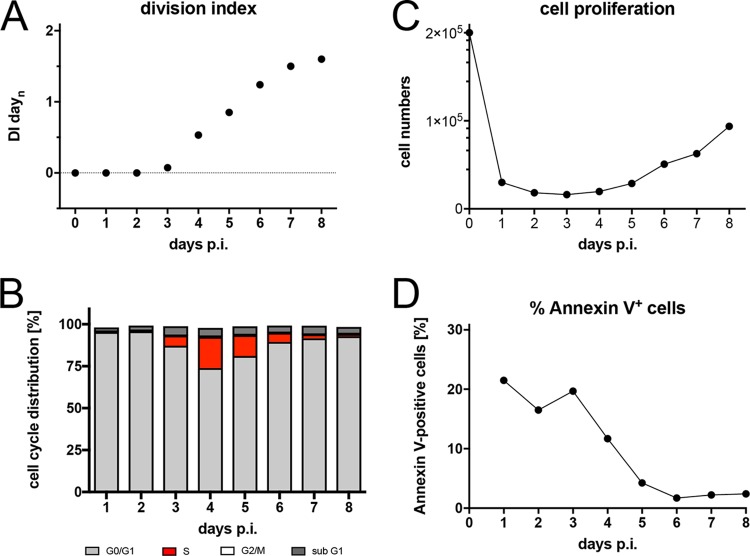
Activation kinetics of naive B lymphocytes cultivated on CD40L feeder cells in the presence of IL-4. As in Fig. 3, naive B lymphocytes isolated from adenoid tissue were loaded with an intracellular dye and cultivated on CD40L feeder cells with IL-4, as described previously (39). (A) Division index is shown. (B) Cell cycle distributions of cultivated cells are provided. (C) Viable cells were counted by flow cytometry. (D) Binding of annexin V was analyzed by flow cytometry. Shown are the results from one representative experiment out of three with B lymphocytes from three different B-cell donors.

Stimulation of human B lymphocytes with CD40 ligand (CD40L) and the cytokine interleukin 4 (IL-4) induces cell activation and unlimited B-cell proliferation independent of EBV infection (38, 39). We reanalyzed this model and found that the kinetics of naive B-cell survival, activation, and proliferation after CD40L and IL-4 stimulation did not differ in principle from EBV-infected resting B lymphocytes in the first week (Fig. 4). In the course of three independent experiments, the fraction of B cells undergoing DNA synthesis peaked on day 4 p.i., but the rate of B-cell proliferation was reduced in this model, which resulted in a slow increase of total cell numbers in the observation period (Fig. 4C). Again, the onset of cellular DNA synthesis took place on day 3 p.i. (Fig. 4B), and the first cell divisions became apparent 1 day later (Fig. 4A).

### EBNA2 is essential for entry into reprogramming.

**FIG 5:**
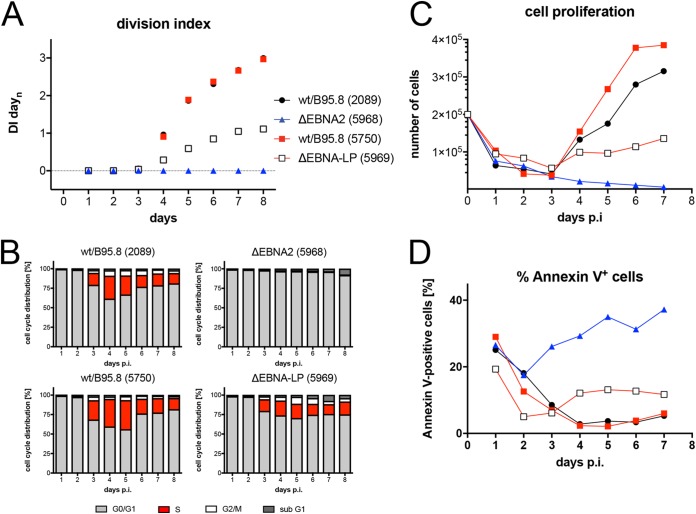
Activation kinetics of naive B lymphocytes infected with mutant EBVs negative for EBNA-LP or EBNA2. As in Fig. 3, sorted naive B lymphocytes were loaded with an intracellular dye and infected with the indicated viruses, which are wild type with respect to viral latent genes [wt/B95.8 (2089) and wt/B95.8 (5750)] or are incapable of expressing EBNA2 [ΔEBNA2 (5968)] or EBNA-LP [ΔEBNA-LP (5969)]. (A) Division index is shown. (B) Cell cycle distributions of cultivated cells are provided. (C) Viable cells were counted by flow cytometry. (D) Binding of annexin V was analyzed by flow cytometry. The results from one representative experiment out of five experiments with B lymphocytes from individual donors are shown.

EBNA2 has been identified to be essential for B-cell immortalization by EBV (5, 6). EBNA2’s functions are also required immediately after EBV infection of B lymphocytes (16) and for the continuous propagation of lymphoblastoid cell lines (7). We revisited EBNA2’s role in the first week of infection with an EBV mutant incapable of translating EBNA2 mRNA, because it carries a mutation of EBNA2’s translational start codon (Table 1). As expected, naive B lymphocytes infected with the ΔEBNA2 (5968) mutant were not activated and did not survive until day 3 p.i. to synthesize cellular DNA (Fig. 5). The data confirm that EBNA2 is central in all early events in B-cell activation and survival *ex vivo*.

### EBNA-LP has auxiliary, supportive functions in B-cell activation and stable latent infection.

The function of EBNA2 is well defined, but the contribution of EBNA-LP to B-cell activation and transformation is less clear. Early experiments with a C-terminally truncated EBNA-LP indicated that its functions might be critical in supporting the initial steps during EBV infection (5). This finding was subsequently confirmed (40), and a recent report suggested that EBNA-LP is an essential gene for the transformation of naive B lymphocytes (41). We generated a pair of recombinant EBVs with six complete copies of EBV’s BamHI-W-repeats, each of which contains the W-exons of EBNA-LP (Fig. S2), very similar to the work of Szymula et al. (41). The EBV recombinant wt/B95.8 (5750) is essentially wild type (Table 1), but its derivative ΔEBNA-LP (5969) is incapable of expressing EBNA-LP because each BamHI-W-repeat carries a translational stop codon in the W1 exon of EBNA-LP (Fig. S2).

10.1128/mBio.01723-19.2 FIG S2 Genetic map of the EBNA-LP and EBNA2 loci of wt/B95.8 (5750) and ΔEBNA-LP (5969) mutant EBV. (A) Details of the two EBV strains, the wt/B95.8 (5750) and ΔEBNA-LP (5969) mutant, engineered to carry six copies of the BamHI-W-repeats are shown. Indicated are the two *cis*-acting elements *oriP* (with its family of repeats and dyad symmetry elements [FR and DS, respectively]) and *oriLyt* and the exons encoding the EBNA-LP gene (W0, [W1, W2]_6_, Y1, and Y2), EBNA2, BHLF1, and BHRF1. The BamHI-W repeats are flanked by XhoI sites, and the BamHI sites preserved in wt/B95.8 (5750) and ΔEBNA-LP (5969) mutant are indicated. Two alternative splicing forms of the bicistronic EBNA-LP/EBNA2 transcripts initiating from either the Cp or Wp promoter are shown below the genetic maps. (B) The schematic composition of the first BamHI-W repeat with parts of its preceding BamHI-C fragment in two EBV strains is shown together with the relevant exons C2, W0, W1/W1′, and W2. The restriction enzyme sites BamHI and BglII are indicated in the EBV strain wt/B95.8 (2089) that are altered in the wt/B95.8 (5750) and ΔEBNA-LP (5969) mutant EBVs. In the ΔEBNA-LP (5969) mutant, each copy of the BamHI-W repeat carries a translational stop codon in the W1 exon indicated by an XbaI site terminating the translation of the EBNA-LP gene. The codon usage in the W1 exon of the wt/B95.8 (5750) and ΔEBNA-LP (5969) mutant EBV strains is provided. Download FIG S2, PDF file, 1.0 MB. Copyright © 2019 Pich et al. 2019 Pich et al. This content is distributed under the terms of the Creative Commons Attribution 4.0 International license.

Sorted naive B lymphocytes were infected with both viruses, and the infected cells were analyzed daily. Cells infected with ΔEBNA-LP (5969) divided less vigorously (Fig. 5A) and showed an increased fraction of annexin V-positive cells (Fig. 5D). DNA synthesis was first detectable on day 3 p.i., but the percentage of S-phase cells remained lower thereafter (Fig. 5B) compared with the two wild-type EBV stocks wt/B95.8 (2089) and wt/B95.8 (5750). The initial loss of B lymphocytes during the first 3 days of infection was comparable (Fig. 5C). The first cell division was clearly detectable on day 4 p.i., but the ΔEBNA-LP (5969)-infected cells did not support the rapid phase of cell proliferation as seen with the wild-type controls (Fig. 5A and C). As a consequence, the number of B cells infected with ΔEBNA-LP (5969) increased very slowly over time.

We also asked if naive B lymphocytes infected with ΔEBNA-LP (5969) can establish stable lymphoblastoid cell lines. From different anonymous donors, we expanded naive B cells infected with ΔEBNA-LP (5969) to considerable cell numbers. We analyzed the protein expression of the established lymphoblastoid cell lines 7 to 8 weeks p.i. and found that the cells did not express EBNA-LP protein as expected (Fig. S3). However, once established, these cells maintained their proliferative phenotype indistinguishably from wild-type EBV-infected lymphoblastoid cell lines. These results mostly recapitulate recently published findings (41) but do not resolve the issue of why EBNA-LP functions are essential in naive B lymphocytes isolated from cord blood (41), whereas the proliferation of the same cell type isolated from adenoid tissue does not depend on EBNA-LP (Fig. S3).

10.1128/mBio.01723-19.3 FIG S3 Steady-state levels of EBNA2 and EBNA-LP proteins in B cells infected with three different EBV strains. Naive B lymphocytes were isolated from adenoid tissue from two different donors and infected with wt/B95.8 (2089), wt/B95.8 (5750), or ΔEBNA-LP (5969) mutant EBV at an MOI of 0.1. Cells were cultivated for 7 (experiment A) or 8 weeks (experiment B) and protein extracts from B cells were analyzed with antibodies specific for EBNA2 or EBNA-LP, as indicated. ΔEBNA-LP (5969) mutant EBV-infected cells did not express EBNA-LP, as expected. The results from two experiments out of three are shown. Download FIG S3, PDF file, 1.9 MB. Copyright © 2019 Pich et al. 2019 Pich et al. This content is distributed under the terms of the Creative Commons Attribution 4.0 International license.

### EBV microRNAs support B-cell reprogramming.

**FIG 6:**
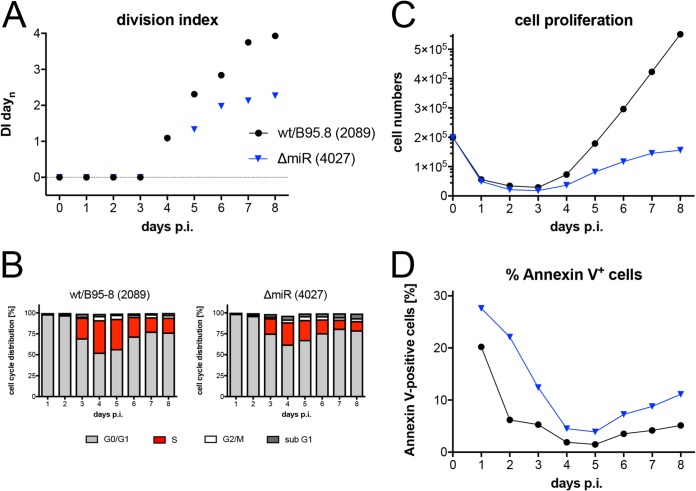
Activation kinetics of naive B lymphocytes infected with an EBV strain lacking all viral miRNAs. As in Fig. 3, sorted naive B lymphocytes were loaded with an intracellular dye and infected with wild-type wt/B95.8 (2089) EBV or ΔmiR (4027) mutant negative for EBV’s miRNAs (42). (A) Division index is shown. (B) Cell cycle distributions of cultivated cells are provided. (C) Viable cells were counted by flow cytometry. (D) Binding of annexin V was analyzed by flow cytometry. The results from one representative experiment out of five experiments with B lymphocytes from five individual donors are shown.

EBV encodes up to 44 viral miRNAs that were found to support the survival of human B lymphocytes in the early phase of infection (42, 43). We reanalyzed the phenotypes of sorted naive B lymphocytes infected with two mutant EBVs that express no viral miRNAs. The results confirmed the previous findings and documented that B cells infected with the ΔmiR (4027) or r_ΔmiR (6338) mutant (Table 1) showed a higher ratio of annexin V-positive cells and accumulated to lower cell numbers in the first 8 days of infection (Fig. 6C and D and S4). The reduced cell numbers were also due to a lower division index (Fig. 6A) and a reduced percentage of S-phase cells (Fig. 6B) compared with their parental wild-type EBV stocks, confirming that EBV’s miRNAs regulate early cell cycle functions (42). Remarkably, the phenotypes observed with naive B cells infected with the two ΔmiR mutants or the ΔEBNA-LP (5969) mutant EBV (Fig. 5) are comparable.

10.1128/mBio.01723-19.4 FIG S4 Analysis of cell proliferation and annexin V binding of B cells infected with mutant EBVs negative for viral noncoding RNAs. Naive B lymphocytes were isolated from adenoid tissue, physically sorted, and infected with four different EBV strains, as indicated. Their genotypes are summarized in Table 1. The cell numbers and the fraction of annexin V-positive cells were analyzed daily. The results from one representative experiment out of four are shown. Download FIG S4, PDF file, 0.1 MB. Copyright © 2019 Pich et al. 2019 Pich et al. This content is distributed under the terms of the Creative Commons Attribution 4.0 International license.

### LMP2A but not LMP1 nor the two noncoding EBERs contribute to the prelatent phase.

**FIG 7:**
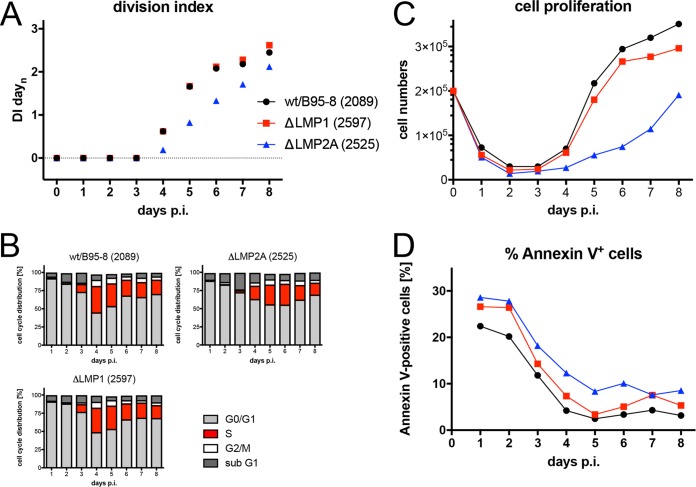
Activation kinetics of naive B lymphocytes infected with an LMP1-negative or LMP2A-negative EBV strain. As in Fig. 3, sorted naive B lymphocytes were loaded with an intracellular dye and infected with the wild-type wt/B95.8 (2089) EBV strain or with ΔLMP1 (2597) mutant EBV, which carries a knockout of EBV’s latent membrane protein 1 [LMP1] (14) or with a ΔLMP2A (2525) mutant EBV with a deletion of the first exon of LMP2A (97). (A) Division index is shown. (B) Cell cycle distributions of cultivated cells are provided. (C) Viable cells were counted by flow cytometry. (D) Binding of annexin V was analyzed by flow cytometry. The results from one representative experiment out of three experiments with B lymphocytes from three individual donors are shown.

Apart from EBNA2, EBNA-LP, and the miRNAs, other latent EBV genes that could play a critical role in the first days of infection include LMP1, LMP2A, EBER1, and EBER2. Their roles in activating and maintaining B-cell proliferation have been studied by many groups, but their early functions are controversial. We infected sorted naive B lymphocytes isolated from several donors with the knockout mutants ΔLMP1 (2597), ΔLMP2A (2525), and ΔEBER (6431) in which both EBER1 and EBER2 were deleted (Table 1) and found reproducible differences only in cells infected with ΔLMP2A (2525) compared with parental EBVs (Fig. 7 and Fig. S4 in the supplemental material, and Table 1). We also investigated a mutant EBV, ΔEBER/ΔmiR (6432), which is deficient in expressing the two viral noncoding RNAs, EBER1 and -2, and all 44 miRNAs, because they might act in combination. Again, the phenotype of B cells infected with the double mutant did not differ from that of the ΔmiR (6338) mutant EBV (Fig. S4), documenting that only the viral miRNAs but not the two longer EBER RNAs play a discernible role in the prelatent phase.

### EBV mutants in EBNA3A and EBNA3C show a proliferative advantage in the prelatent phase.

**FIG 8:**
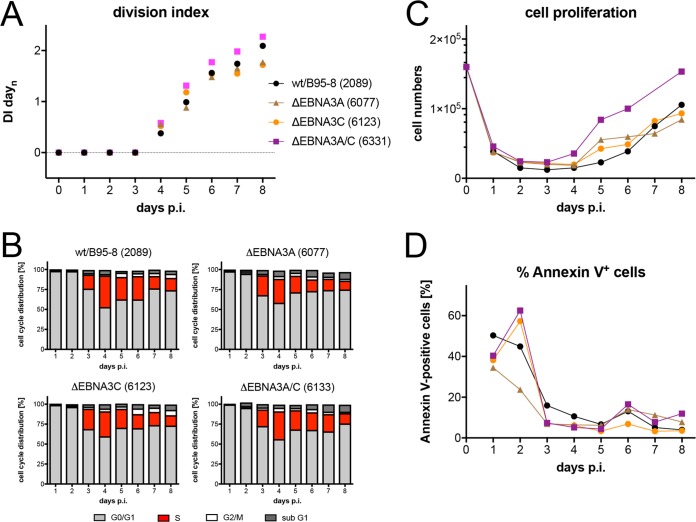
Activation kinetics of naive B lymphocytes infected with EBV strains incapable of expressing EBNA3A, EBNA3C, or both EBNA3 family members. As in Fig. 3, sorted naive B lymphocytes were loaded with an intracellular dye and infected with a wild-type strain [wt/B95.8 (2089)] or three EBV strains deficient in EBNA3A, EBNA3B, or both, as indicated (Table 1). (A) Division index is shown. (B) Cell cycle distributions of cultivated cells are provided. (C) Viable cells were counted by flow cytometry. (D) Binding of annexin V was analyzed by flow cytometry. The results from one representative experiment out of three experiments with B lymphocytes from three individual donors are shown.

The early functions of EBNA3A and EBNA3C have been reported (44, 45), but we wished to include these two latent genes in our study to explore the phenotypes of three single and double EBNA3A and EBNA3C mutant EBVs (Table 1). With knockout EBVs devoid of EBNA3A (6077) or EBNA3C (6123), we found no substantial differences from the parental wild-type EBV (Fig. 8) but noticed slightly higher cell numbers at 5 to 6 days p.i. This finding was puzzling because EBNA3A was found important for inducing the expression of antiapoptotic genes of the cellular host (44), whereas EBNA3C was reported to counteract an antiviral DNA damage response early in EBV-infected B cells (45). This inconsistency with published work led us to engineer and test an EBV mutant deficient in expressing both viral genes. In several experiments with sorted naive B lymphocytes from different donors, the ΔEBNA3A/C (6331) mutant induced a slightly more robust proliferation and fewer annexin V-positive cells than did its parent (Fig. 8).

EBNA3C was reported to prevent a substantial DNA damage response (DDR) observed in the early phase of infection during cellular hyperproliferation in EBV-infected human B lymphocytes (45, 46). Given our findings so far, we wondered if the apoptotic loss of cells during the first 3 days of infection or later phenotypes might stem from this type of antiviral stress response that EBV-infected B cells presumably experience prior to establishing viral latency. In naive B lymphocytes infected with wt/B95.8 (2089) EBV, we analyzed the phosphorylation of the histone variant H2A.X at Ser 139 (γ-H2A.X) by flow cytometry over time. In addition to its well-established role in the DDR for recognition and repair of double-strand breaks, phosphorylation of H2A.X can also be triggered by DNA replication stress (DRS) during the surveillance of ongoing DNA replication in the absence of physical DNA damage (47, 48).

**FIG 9:**
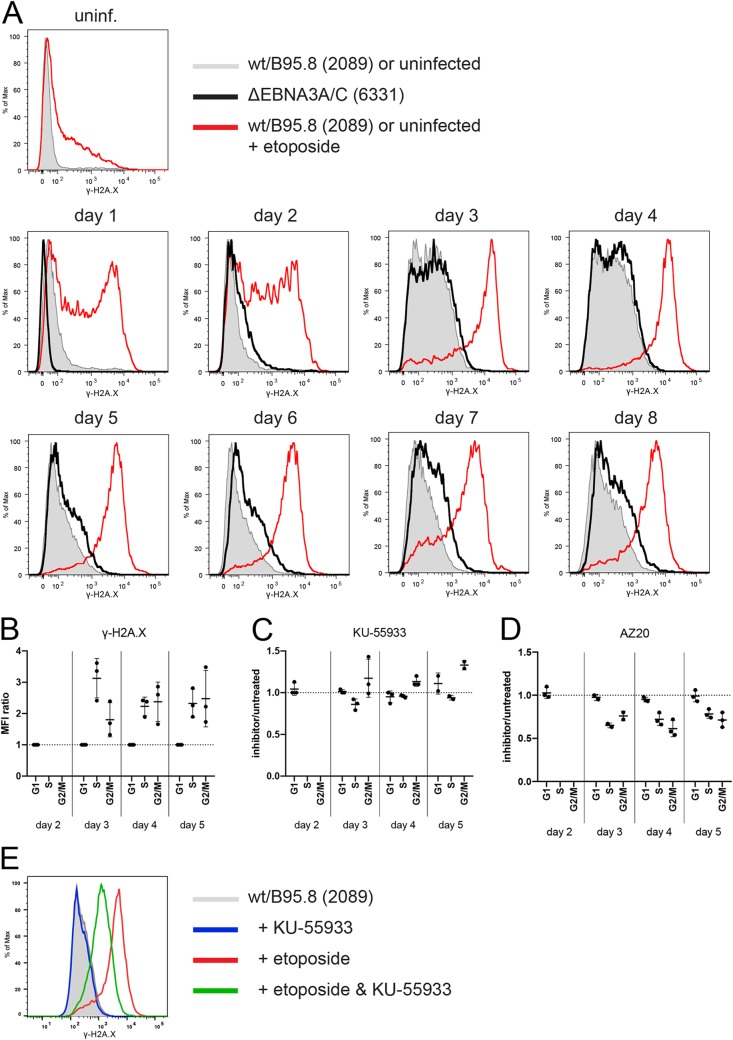
γ-H2A.X levels in uninfected B lymphocytes and cells infected with wild-type EBV or an EBNA3A/3C double-knockout EBV with ATM and ATR inhibitors. (A) FACS-sorted naive B lymphocytes were infected with wt/B95.8 (2089) EBV or ΔEBNA3A/C (6331) mutant EBV with an MOI of 0.1 and analyzed at different time points, as indicated. Intracellular staining detected the levels of the phosphorylated histone variant H2A.X (γ-H2A.X) in viable cells (according to scatter criteria by flow cytometry). As a positive control, uninfected B lymphocytes or cells infected with wt/B95.8 (2089) EBV were treated with 85 μM etoposide for 1 h prior to harvest (red line). The results from one representative experiment out of three with B lymphocytes from three individual donors are shown. (B) FACS-sorted naive B lymphocytes were infected with B95.8 EBV with an MOI of 0.1. The cells were pulse labeled with EdU (5-ethynyl-2′-deoxyuridine, a nucleoside analog to thymidine incorporated into DNA during active DNA synthesis) for 1 h, fixed, permeabilized, and frozen at –80°C at the indicated time points. For flow cytometry analysis, the cells were thawed and analyzed after intracellular staining with an antibody directed against γ-H2A.X and a click reaction between EdU and Alexa Fluor 488. Single viable cells were considered by gating, and the mean fluorescence intensity (MFI) values of the fluorochrome-coupled γ-H2A.X specific antibody were separately recorded in the cell cycle fractions G_1_, S, and G_2_/M. MFI values of the S and G_2_/M fractions were compared with the MFI values of G_1_ cells and expressed as ratios of S versus G_1_ or G_2_/M versus G_1_ MFI levels. The MFI levels of γ-H2A.X fluorescence in G_1_ were set to 1.0. On days 3 to 5, cells in S and G_2_/M had 2- to 3-fold higher γ-H2A.X levels compared to those with cells in G_1_. Cells infected for 2 days do not cycle or synthesize DNA. The results from three independent experiments are summarized. (C) B95.8-infected B lymphocytes were incubated with EdU together with the ATM inhibitor KU-55933 or were incubated with EdU only for 1 h prior to harvest, as in panel B. Shown are the MFI ratios of γ-H2A.X levels in the different cell cycle fractions of KU-55933-treated versus untreated cells. KU-55933 showed a slight inhibitory effect on day 3 p.i. in S-phase cells, but other γ-H2A.X levels were not reduced but sometimes even elevated when KU-55933 was applied. The results from three independent experiments are summarized. (D) The experimental setup is identical to that in panel C except that the ATR inhibitor AZ20 was applied for 1 h together with EdU. Cells in S and G_2_/M phase but not cells in G_1_ phase showed a clear reduction in γ-H2A.X levels in the presence of AZ20 on days 3, 4, and 5 p.i. The results from three independent experiments are summarized. (E) The histogram demonstrates the inhibitory effect of the inhibitor KU-55933 on an etoposide-induced DDR. B lymphocytes were infected with wt/B95.8 (2089) EBV for 5 days and treated with KU-55933 for 1 h prior to analysis with the γ-H2A.X specific antibody by intracellular flow cytometry (red line in the histogram). Concomitant addition of the ATM inhibitor KU-55933 for 1 h together with etoposide reduced the induced levels of γ-H2A.X considerably (green), whereas the addition of KU-55933 alone (blue) had no effect on cells that were not treated with etoposide (gray-shaded histogram).

Levels of γ-H2A.X were very low in sorted naive and uninfected B lymphocytes and in intact cells infected for the first 2 days (Fig. 9A). On days 3 and 4 p.i., the global levels of γ-H2A.X increased considerably in the population of viable cells and decreased again on day 5 p.i. and later (Fig. 9A). Interestingly, short-term incubation with etoposide, a potent inducer of DDR, led to an increase in γ-H2A.X staining in uninfected cells and an even further increase in infected cells, indicating that the potential of EBV-infected cells to respond to DNA damage is intact in the prelatent phase. We also analyzed B cells infected with the ΔEBNA3A/C (6331) mutant. The cells showed marginally higher levels γ-H2A.X staining than did wild-type EBV-infected cells starting on day 6 p.i. (Fig. 9A), but clearly no major shift, as seen after short-term etoposide induction. Increased phosphorylation levels of H2A.X paralleled the onset of DNA synthesis on day 3 p.i. (Fig. 8B) and remained equally high during the initial, very rapid cell divisions on day 4; but they were lower on the following days, suggesting that γ-H2A.X staining reflects DRS rather than the cells’ response to DNA damage (49).

Both genotoxic and nongenotoxic stresses interrupt the p53-MDM2 loop to stabilize p53 increasing its steady-state protein level. This process leads to changes in the expression of hundreds of p53-responsive genes, including the checkpoint inhibitor *WAF1-CIP1* coding for p21, and induces a halt in proliferation preventing the transmission of damaged DNA to daughter cells. We looked at the protein levels of both p53 and p21 as well as Ku70 and Rad51, which are commonly upregulated during DDR. We found considerable levels of p53 and p21 in wild-type EBV-infected cells starting on day 4 p.i. and onwards (Fig. S5); in addition, levels of Ku70 and Rad51 also increased in parallel with p53 and p21. Unexpectedly, in B cells infected with the ΔEBNA3A/C (6331) mutant, we barely detected p53 protein and found slightly reduced levels of p21 compared with wt/B95.8 (2089)-infected cells (Fig. S5). Levels of Rad51 appeared to be marginally reduced in cells infected with the ΔEBNA3A/C (6331) mutant, whereas Ku70 levels seemed to be similar in cells infected with the ΔEBNA3A/C (6331) mutant or wt/B95.8 (2089) EBV. The findings suggest that cells infected with the EBNA3A/EBNA3C double-mutant EBV might experience lower stress levels, which could explain their proliferating more vigorously than wild-type EBV-infected B lymphocytes in the prelatent phase (Fig. 8).

10.1128/mBio.01723-19.5 FIG S5 Western blotting of proteins regulated during cellular DNA damage response. Uninfected human primary B lymphocytes (uninf.) and cells infected with wt/B95.8 (2089) EBV or ΔEBNA3A/C (6331) mutant EBV were harvested at the indicated time points (days p.i.). Protein lysates of 5 × 10^5^ cells per lane were loaded, and the steady-state levels of the indicated proteins were analyzed with antibodies directed against p53, p21, Ku70, or Rad51. An EBNA2-specific antibody was used to monitor the onset of EBNA2 expression. Lysates obtained from 293T cells incubated with 85 μM etoposide for 1 h were loaded as control (cont). The results from one experiment out of two are shown. Download FIG S5, PDF file, 1.8 MB. Copyright © 2019 Pich et al. 2019 Pich et al. This content is distributed under the terms of the Creative Commons Attribution 4.0 International license.

### In the prelatent phase, EBV infection does not induce a DNA damage response but causes DNA replication stress.

We were puzzled by these unexpected findings because they are in obvious conflict with previous reports (45, 46). To discriminate between DDR and DRS as the confounding source of the elevated γ-H2A.X signal, we asked in which phase of the cell cycle the infected cells show an increase in γ-H2A.X levels. We reasoned that if DRS is the major driver of this effect, it should only increase γ-H2A.X levels in S-phase and postreplicative G_2_/M cells, whereas DDR would equally affect all cells independent of the cell cycle state. We infected sorted naive B lymphocytes from adenoid tissue with the reference strain B95.8 and an MOI of 0.1 and labeled the newly synthesized cellular DNA with EdU (5-ethynyl-2′-deoxyuridine), a nucleoside analog to thymidine, for 1 h prior to analysis. We found that cells in S and in G_2_/M phases showed a 2- to 3-fold higher level of γ-H2A.X staining, whereas G_1_-phase cells were unaffected (Fig. 9B). To further support our hypothesis, we combined this analysis with two inhibitors, KU-55933 (Fig. 9C) (50) or AZ20 (Fig. 9D) (51), known to specifically block the activation of ataxia telangiectasia-mutated (ATM) and ATR (ATM and rad3 related), the two major DNA damage checkpoint kinases responsible for γ-H2A.X phosphorylation. Whereas ATM is primarily activated by DDR, ATR responds to stretches of RPA-coated single-stranded DNA (ssDNA) at stalled replication forks during DRS (52). While KU-55933 clearly repressed an etoposide-induced DDR signal in EBV-infected B cells (Fig. 9E), it did not reduce the mean fluorescence intensity (MFI) of γ-H2A.X staining in EBV-infected cells on days 3 and 4 p.i. but merely increased it in S- and G_2_/M-phase cells (Fig. 9C). In contrast, AZ20 caused a considerable reduction in γ-H2A.X levels in all actively cycling cells on days 3 to 5 (Fig. 9D), indicative of a reduction in the DRS as the primary source of the elevated γ-H2A.X signal.

### EBNA1 does not contribute to B-cell activation, cell cycle entry, or early cell proliferation.

**FIG 10:**
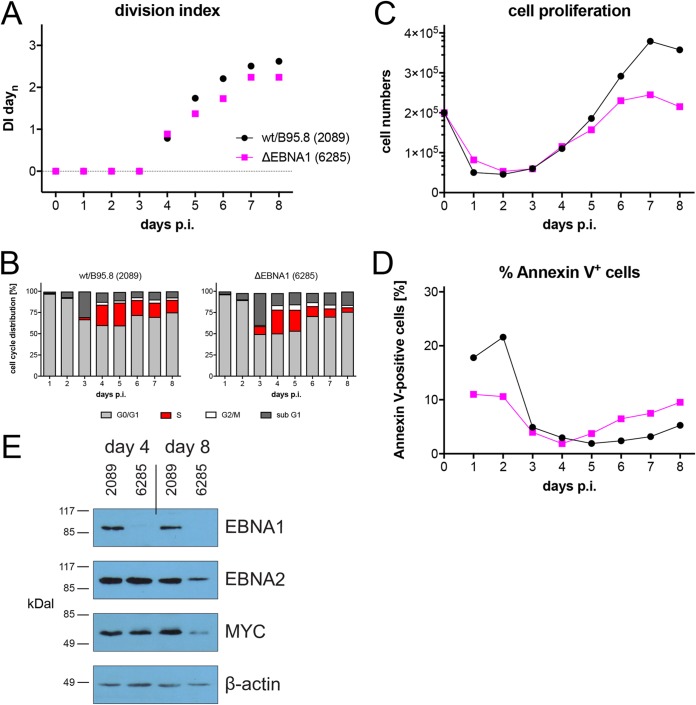
Activation kinetics of naive B lymphocytes infected with an EBV strain lacking EBNA1. As in Fig. 3, sorted naive B lymphocytes were loaded with an intracellular dye and infected with wild-type wt/B95.8 (2089) EBV or the ΔEBNA1 (6285) mutant, which cannot express EBNA1 due to a point mutation in EBNA1’s translational start codon. (A) Division index is shown. (B) Cell cycle distributions of cultivated cells are provided. (C) Viable cells were counted by flow cytometry. (D) Binding of annexin V was analyzed by flow cytometry. (E) Steady-state protein levels of B cells infected with wtB95.8 (2089) or ΔEBNA1 (6285) mutant EBV for 4 or 8 days were analyzed by Western blotting immunodetection with antibodies directed against EBNA1, EBNA2, MYC, or β-actin. The results from one representative experiment out of five are shown.

Next to EBNA2, EBNA1 is the second latent protein, whose functions have been studied intensively. EBNA1 is critical for the extrachromosomal maintenance of genomic EBV DNA, and it acts as a transcription factor regulating the expression of viral and cellular genes in latently EBV-infected cells. We asked whether this viral factor makes important contributions during early infection and engineered a viral mutant in which the translation of EBNA1’s open reading frame is disabled. Sorted naive B lymphocytes were infected with this mutant EBV, termed ΔEBNA1 (6285), or with the reference wild-type strain wt/B95.8 (2089). Surprisingly, the phenotypes of B cells infected with this pair of viruses barely differed (Fig. 10). Cells infected with the ΔEBNA1 (6285) mutant or wt/B95.8 (2089) EBV underwent similar rates of initial death, became similarly activated, started cellular DNA synthesis on day 3, and began with rapid cell divisions on day 4 p.i. as we had observed with all mutant EBVs but the ΔEBNA2 (5968) mutant so far (Fig. 10). Proliferation of B cells infected with the ΔEBNA1 (6285) mutant slowed down starting on day 6 or 7 p.i. (depending on the donor’s B cells) but showed only a slight increase in annexin V-positive cells (Fig. 10C and D). We analyzed the protein levels of EBNA2, EBNA1, and MYC and found that cells infected with the ΔEBNA1 (6285) mutant expressed no EBNA1, as expected, but comparable levels of EBNA2 and MYC protein on day 4 p.i. (Fig. 10E). On day 8 p.i., however, the levels of both EBNA2 and MYC were considerably lower than wild-type EBV-infected cells, but B cells infected with the ΔEBNA1 (6285) mutant survived even longer, shrank in volume (Fig. S6), and became extinct about 2 to 3 weeks postinfection. Whereas EBNA1 plays a central role in the maintenance of EBV latency, our data indicate that EBNA1 is absolutely dispensable for the early stage of EBV-infected primary naive B cells.

10.1128/mBio.01723-19.6 FIG S6 Flow cytometry-based cell size analysis of B lymphocytes infected with ΔEBNA1 (6285) mutant or wt/B95.8 (2089) EBV. Human primary B cells from adenoids were infected with wt/B95.8 (2089) or ΔEBNA1 (6285) mutant EBV with an MOI of 0.1 and analyzed by flow cytometric analysis, according to forward-scatter (FSC-A; *x* axis) and side-scatter (SSC-A; *y* axis) criteria at the indicated time points. Viable cells are surrounded by the indicate gate (polygonal magenta line). Cells infected with the two EBV strains similarly gain in size and granularity until 8 days p.i., but B cells infected with ΔEBNA1 (6285) mutant EBV showed a decrease in volume starting on day 10 p.i., and the main population of cells became very small 2 weeks p.i. compared with cells infected with wt/B95.8 (2089) EBV. Shown are the results from one representative experiment out of three. Download FIG S6, PDF file, 0.7 MB. Copyright © 2019 Pich et al. 2019 Pich et al. This content is distributed under the terms of the Creative Commons Attribution 4.0 International license.

### A minimal set of EBV genes is sufficient to support B-cell activation and proliferation.

So far, we learned that many viral latent genes are dispensable in EBV-infected B cells during the prelatent phase. We wondered if the expression of only EBNA2, EBNA-LP, and BHRF1 might be sufficient to activate primary B lymphocytes and drive them into proliferation. Toward this end, we revisited the two EBV plasmids p554 and p613 we had engineered earlier (5). They both encompass a contiguous fragment of EBV with the nucleotide coordinates 7315 to 56083 of the wt/B95.8 strain. This fragment carries *oriP*, EBNA-LP, and EBNA2 but only two complete copies and a truncated copy of the BamHI-W-repeat element. This EBV DNA fragment also contains the lytic origin of DNA replication, *oriLyt*, together with the BHRF1 and BHLF1 loci, which flank it. The plasmid p554 and p613 DNAs can be packaged into infectious EBV particles, because p554 and p613 also contain terminal repeats (TRs), the essential DNA packaging elements of EBV (5, 53). p554 and p613 differ in that p613 is incapable of expressing EBNA2 (5).

**FIG 11:**
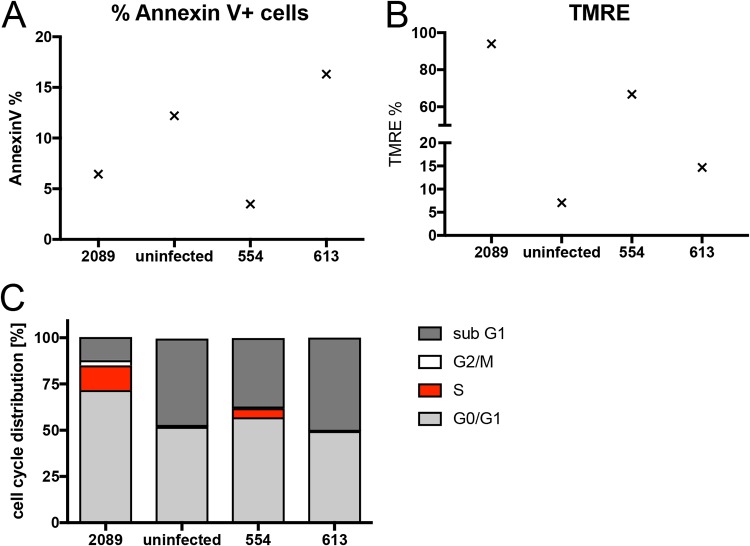
A vector approach identifies EBNA2 as an essential viral gene in EBV’s prelatent phase. Naive primary B lymphocytes were infected for 6 days with vector stocks obtained by packaging the plasmids p554 and p613 (5) into EBV-based viral particles (54). Noninfected B lymphocytes and cells infected with an MOI of 0.1 of wt/B95.8 (2089) virus stock served as negative and positive controls, respectively. (A) Annexin V binding and (B) TMRE staining of active mitochondria indicated apoptotic and metabolically active cells, respectively. (C) BrdU incorporation revealed cycling cells in S phase. The results from one representative experiment out of three are shown. p554 encodes EBNA2, EBNA-LP, BHRF1, and BHLF1, whereas p613 lacks EBNA2.

We separately introduced the two plasmids into EBV particles with the help of a packaging cell line that harbors a nontransforming EBV helper virus genome with several genetic modifications that include a deleted EBNA2 locus (54). Concentrated vector stocks with p554 or p613 were used to infect naive B lymphocytes and living cells (according to forward- and side-scatter criteria in flow cytometry) were analyzed for their annexin V binding, tetramethylrhodamine, ethyl ester (TMRE) staining (a marker of active mitochondria), and BrdU incorporation (Fig. 11). The vector stocks did not nearly reach EBV titers that were used in all the previous experiments (up to 4 × 10^6^ GRU/ml), but B cells infected with p554 clearly were alive on day 6 p.i. (Fig. 11). Only a small percentage of the living cells bound annexin V (Fig. 11A), but a high fraction stained with TMRE, indicating robust mitochondrial activity (Fig. 11B). These observations were in contrast to cells incubated with p613 vector stocks devoid of EBNA2 (Fig. 11B). At a much lower level than wt/B95.8 (2089) EBV-infected cells, B cells infected with p554 vector stocks incorporated BrdU, indicating that they underwent DNA replication, mitosis, and cell divisions (Fig. 11C). On day 6 p.i., few intact B cells infected with p613 vector stocks or noninfected cells survived, which did not cycle (Fig. 11C). This experiment confirms that EBNA2 is the essential viral factor in the prelatent phase of human B cells and suggests that it probably in conjunction with EBNA-LP is sufficient to reprogram resting human B lymphocytes.

## DISCUSSION

### Reconstruction of a B95.8-based EBV field strain.

In this study, we present a version of the B95.8 strain of EBV (29) in which we reverted the ∼12-kb deletion present in this laboratory isolate (32). Recently, another group has followed this approach (55) using a fragment from the Akata strain of EBV. The authors demonstrated that the miRNAs absent in B95.8 were expressed in cells infected with the reconstituted virus. They also showed a downregulation of the *NDRG1* gene expression in epithelial cells infected with the reconstituted virus compared to cells infected with their recombinant parental B95.8 strain (55). We employed our bacterial artificial chromosome (BAC)-derived wt/B95.8 (2089) strain to obtain a virus that is as close as possible to a B95.8-based field strain and to allow further engineering and reverse genetic experiments. B lymphocytes infected with the reconstituted strain r_wt/B95.8 (6008) or the parental wt/B95.8 (2089) strain behave very similarly in our proliferation experiments (Fig. 3), but we suspect that the presence of all BART miRNAs in r_wt/B95.8 (6008) will lead to observable phenotypes in other infection models. As we have shown recently, the viral miRNAs play a remarkable role in inhibiting the T-cell recognition of EBV-infected cells *in vivo* and *in vitro* (56–58). It will be interesting to compare both related viruses in, e.g., humanized mice that may reveal important additional roles of the BART miRNAs encoded in the r_wt/B95.8 (6008) strain of EBV only.

### Limitations of the *in vitro* B-cell infection model.

One of the major problems in this field is the lack of standardization of assays and the absence of a commonly accepted nomenclature to describe the importance of latent viral genes in *in vitro*-infected B cells. As pointed out in the introduction, discrepant studies can even devolve into semantic debates: is a gene essential or critical and what is the difference? In Table 2, we attempt to clearly define three terms summarizing the effects of individual latent EBV genes on initial cell survival and proliferation during the first 8 days in the prelatent phase and on the outgrowth of lymphoblastoid cells during the first 4 weeks of infection. We also consider the long-term maintenance of the LCL phenotype with respect to the literature in Table 2.

**TABLE 2:** *In vitro* phenotypes of EBV’s individual latent genes in the different phases of B-cell infection^*a*^

EBV gene	Gene role by phase			Reference(s) or source
	In the prelatent phase of infection (up to day 8 p.i.)	During outgrowth of lymphoblastoid cells (up to 4 wk p.i.)	In established lymphoblastoid cell lines (LCLs)	
EBNA1	Dispensable	Critical	Dispensable^*b*^	This study, 59, 98
EBNA-LP	Critical	Critical/Dispensable	Dispensable	This study, 5, 40, 41
EBNA2	Essential	Essential	Essential	This study, 5, 6, 16
EBNA3A	Dispensable	Dispensable	Dispensable	This study, 9, 15, 17, 45, 99, 100
EBNA3B	Not studied	Not studied	Dispensable	101
EBNA3C	Dispensable	Essential^*c*^	Essential^*d*^	This study, 19, 45, 99, 102
LMP1	Dispensable	Critical	Critical^*e*^	This study, 11, 14
LMP2A	Critical	Critical/dispensable	Critical/dispensable	This study, 103, 104
LMP2B	Not studied	Not studied	Dispensable	103, 105
EBERs	Dispensable	Dispensable	Dispensable	This study, 106, our unpublished data
miRNAs	Critical	Critical/dispensable	Dispensable	This study, 42, 43

a Definitions of our nomenclature describing the contributions of EBV’s latent genes in the three different phases of *in vitro* infection are as follows: “essential” indicates that the gene is absolutely required for the survival of the infected B cells, “critical” indicates that the gene contributes detectably to their fitness as well as survival, and “dispensable” indicates that the gene has no apparent phenotype in the EBV-infected B cells.

b In LCLs infected with a ΔEBNA1 EBV mutant, viral DNA can integrate into chromosomes of the cellular host, which is rare but yields stable LCLs.

c As reported by Skalska et al. (19), we see no discernible phenotype in ΔEBNA3C EBV-infected B cells prior to weeks 4 to 6 p.i. (our unpublished data).

d Dispensable in B cells with nonfunctional *CDKN2A* (p16^INK4A^) or Rb protein (17).

e LCLs infected with a ΔLMP1 mutant EBV can be cultivated long term under conditions of high cell density (our unpublished data) or on fibroblast feeder cells (14).

**FIG 12:**
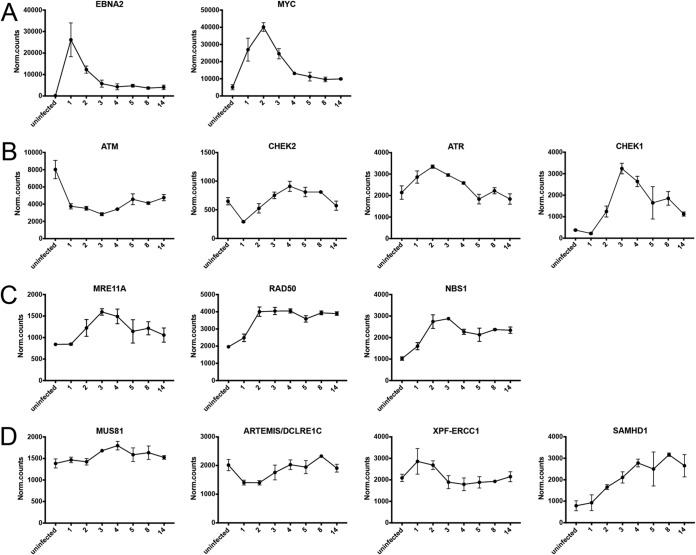
Transcriptional regulation of EBNA2 and selected cellular genes involved in DNA damage response or DNA replication stress. Primary naive B lymphocytes were isolated and infected with wt/B95.8 (2089) EBV. The transcriptomes of uninfected and infected B cells were investigated by RNA sequencing (RNA-seq) technologies at the indicated time points, as described previously (27). (A) EBNA2 and MYC appear to be coregulated. (B) The transcriptional regulation of the ATM-Chek1 (Chk1) and ATR-Chek2 (Chk2) pathways, which are activated by DNA double-strand breaks and single-stranded DNA, respectively, is shown. (C and D) The transcriptional regulation of components of the MRN complex and genes involved in double-strand break (C) and single-strand DNA break and repair (D) are shown. The data were obtained from our recent work (27) and are freely accessible online (http://ebv-b.helmholtz-muenchen.de/).

When we started our experimental work, we did not expect to learn that EBNA2 is the only viral latent gene product that is essential (and presumably also sufficient) to activate resting human primary B cells and to induce their cycling. EBNA-LP, LMP2A, and the viral miRNAs contribute to this process, but naive B lymphocytes infected with mutant EBVs that do not express these individual auxiliary genes readily give rise to lymphoblastoid cell lines. In this experimental model, even EBNA1 seems to be dispensable for several days, but cells infected with an EBNA1-negative EBV only very rarely yield stable cell lines in which the EBV genome is then found to be chromosomally integrated (59). We also learned that the newly infected cells express very high levels of EBNA2 initially (Fig. 12A) (27), which now appear sufficient to reprogram the infected cells (this study) and upregulate mitochondrial one-carbon metabolism (60) until other latent viral gene products support and stabilize the reprogrammed cells long term. Such a secondary role has been proposed for LMP1 (61). LMP1’s mRNA is expressed at substantial levels early on (27) (see also http://ebv-b.helmholtz-muenchen.de/), but an LMP1-negative EBV mutant did not result in a distinct phenotype in the prelatent phase (Fig. 7), confirming recent reports (61, 62).

We found that the ratio of EBV versus its target cell is critical and can even appear toxic at high doses (Fig. 1). At our standard dose (MOI of 0.1 as determined by infecting Raji cells), we can safely assume that each B lymphocyte is infected with at least one biologically active EBV particle on average, given our previous investigations with Daudi cells (26). A higher fraction of B lymphocytes can be identified initially to be EBNA2 positive, when the virus dose is increased (Fig. 1C), but the effect is counterproductive and reduces the number of latently infected cells 8 days p.i. (Fig. 1A). An MOI of 3.0, which equals at least 30 biologically active virions per B lymphocytes, is acutely toxic to the cells (Fig. 1B), suggesting that many copies of genomic EBV DNA lead to a high gene dose of EBNA2, which seems to be detrimental. Very high levels of EBNA2 protein likely result in extreme MYC levels that induce alternative reading frame (ARF) expression (63), which in turn activates p53 and leads to cellular senescence, cell cycle arrest, and apoptotic death (64). This working hypothesis seems plausible but needs to be tested.

The prominent function of EBNA2 in this *in vitro* infection model has been known for a long time, but EBNA2 is rarely found expressed in cells *in vivo* (65). Even EBV field strains in which the EBNA2 gene is deleted have been identified in latently infected Burkitt lymphoma cells, a rare finding (66, 67). How these EBV strains evolve is speculative, but it is likely that the deletion of EBNA2 is a late event in these cells, which are under the control of the *MYC* oncogene translocated into an active immunoglobulin locus.

In mice reconstituted with functional components of a human immune system, it is possible to study EBV’s biology *in vivo* (68), but steps early after infection are difficult to investigate in this tractable model. To our knowledge, an EBNA2-deleted virus has not been studied in this animal model yet, but unexpectedly, EBV strains incapable of expressing EBNA3C were found to establish latency and cause B-cell lymphomas, albeit at a reduced frequency compared with wild-type EBV (69, 70). EBNA3C is a transcriptional repressor and has been found to abrogate the expression of cell cycle inhibitors such as p16^INK4A^ encoded by *CDKN2A*, which is a prerequisite to establish lymphoblastoid cell lines with EBNA3C-deleted EBVs *in vitro* (17–19). Our ΔEBNA3C (6123) mutant EBV did not reveal an early phenotype compared with wild-type EBV-infected B lymphocytes, and the cells showed robust activation and proliferation in the prelatent phase (Fig. 8). B cells infected with EBNA3C deleted mutant EBVs ceased to proliferate about 2 weeks postinfection (19), very much in contrast to the situation *in vivo*. In humanized mice (hu-mice), EBNA3C-deleted viruses established latent infection in B cells while the cells expressed appreciable levels of p16 (69, 70). Possibly, there are several reasons that may lead to the observed differences, but the comparison demonstrates the limitations of the experimental models with which we can study the biology of EBV infection *in vitro*.

Some of these limitations also became obvious in our experiments. Naive primary B lymphocytes isolated from many different donors showed certain unstable phenotypes when infected with EBV. For example, in the experiments shown in Fig. 1, 3, and 5, the numbers of B cells from two donors infected with different wild-type EBV strains exceeded the initially infected and seeded cell numbers on day 8 p.i. The examples shown in Fig. 8 and S1 illustrate that B cells from two other donors showed a less robust recovery with respect to cell numbers when infected with wt/B95.8 (2089) EBV. In almost all settings, at least three independent experiments with B cells from different donors were conducted to validate the experimental outcome with the mutant EBVs, but individual experiments showed a certain variability in particular with respect to cell numbers in the time course experiments. We have no firm clue about the molecular basis of this observation but believe that the experiments reflect both technical as well as donor-specific conditions.

### The master regulator EBNA2.

Several arguments underscore EBNA2’s prominent role in the prelatent phase of infected primary B cells. EBNA2 is a viral enhancer factor and a potent inducer of the *MYC* gene (7, 71). MYC is the master regulator of all cells and controls cellular metabolism, cell growth and differentiation, and cell cycle progression, but it also governs apoptosis and cell death. It is a potent oncoprotein that is highly expressed in Burkitt lymphomas. High levels of MYC protein induce cellular growth, cell cycle entry, and cell proliferation, but, as a consequence, also cause replication stress (72). Induced overexpression of *MYC* increases the fraction of S-phase cells and accelerates S-phase progression probably by initiating premature origin firing. This leads to a higher density of active origins of DNA replication, which are associated with asymmetrical fork progression and stalled replicative DNA intermediates (73). The very high initial expression of EBNA2 already on day 1 p.i. (Fig. 12A) (27) probably causes the timely expression of *MYC* to reach its highest level on day 2 p.i. (Fig. 12A), which in turn explains most of the phenotypic changes we observed during the first days of EBV-infected B cells.

EBNA2 directly targets *MYC* and activates its expression (74). EBNA2 is known to bind to two B-cell enhancers about 500 kbp upstream of the *MYC* gene and brings them in contact with the *MYC* promoter element to achieve its transcriptional activation (71). EBNA2 was also identified to be the key component of this enhancer complex together with additional cellular and viral factors such as NF-κB subunits and EBNA-LP, respectively (75). To our knowledge, there are no data that describe the action of EBNA2 at these *cis*-acting elements during the very first days of infection, but our data suggest that EBNA2 acts on the *MYC* locus and its upstream enhancers initially, when EBV infects quiescent primary B lymphocytes.

### Does infection with EBV induce a genotoxic signal in primary B cells?

When cellular machines sense damaged DNA, they induce a DNA damage response (DDR) and initiate DNA repair to prevent genetic instability of the cell. Similarly, during cellular DNA replication, replication forks can stall when they encounter sequences that are intrinsically difficult to replicate or when the cell experience genotoxic stress during mitosis. These apparent problems of cells progressing through the S phase of the cell cycle are generally referred to as DNA replication stress (DRS). Many cellular factors are known that manage DDR and/or DRS to maintain the genetic integrity of the cell or to induce its subsequent death. Using our recent data set (27), we asked whether EBV infection regulates these factors in the prelatent phase to evaluate if they play a decisive role in the reprogramming and survival of the virally infected B cells. It is also obvious from these data that EBV infection regulates the gene expression of the ATM-Chk2 and ATR-Chk1 pathways, which are induced by DNA double-stranded breaks and exposed single-stranded DNA and cope with DDR and DRS, respectively (Fig. 12B). In particular, Chk1 is dramatically induced starting on day 2 p.i. (Fig. 12B). The ATR-Chk1 pathway is active during DNA synthesis and acts primarily as a replication checkpoint.

The trimeric MRN complex, which consists of MRE11A, RAD50, and NBS1 (also called Nibrin [NBN]), is a multifunctional DDR machine (76). Together with other damage recognition receptors, the MRN complex can detect damaged DNAs to initiate DNA repair signals, but it can also start a cascade of innate immune signals when certain viral DNAs are detected. Not surprisingly, DNA viruses such as adenovirus (77) target the MRN complex to prevent its functions during lytic infection with this pathogen, which induces a strong DDR signal when the amplified viral DNA accumulates (78). EBV does not start its lytic phase initially as adenoviruses do (25), but EBV infection upregulates the transcript levels of all three components of the MRN complex 2- to 3-fold as early as on day 2 (Fig. 12C) and prior to the onset of cellular DNA synthesis 1 day later. The levels of RAD50 transcripts remain unchanged thereafter, whereas MRE11A and NBS1 are slightly reduced when established lymphoblastoid cells emerge (Fig. 12C). It thus appears that these three proteins are upregulated prior to or concomitant with the first occurrence of DNA replication, but their mRNA levels remain constant or are reduced later when the cells start to proliferate rapidly on days 4 and 5 p.i.

The endonucleases, MUS81, ARTEMIS (DCLRE1C), and XPF-ERCC1, act on stalled replication forks to resolve DRS during the S and G_2_ phases and/or during mitosis and cleave single-stranded DNAs (79). In the prelatent phase, the transcript levels of these important enzymes vary modestly, with the exception of XPF-ERCC1, which peaks on days 1 and 2 p.i. (Fig. 12D). In contrast, EBV infection induces SAMHD1, which promotes the degradation of nascent strand DNA at stalled replication forks by binding to and stimulating the exonuclease activity of MRE11A, which is upregulated to reach maximal levels on day 3 p.i. (Fig. 12C). MRE11A degrades nascent strand DNA during fork resection and thus facilitates fork restart. Transcript levels of SAMHD1 increase over time and stay elevated when the cells undergo rapid rounds of cell division and DNA replication (Fig. 12D), suggesting that high levels of both MRE11A and SAMHD1 limit DRS (80) in this phase of viral infection and later when lymphoblastoid cell lines emerge. On the other hand, the enzymatic dNTPase activity of SAMHD1 was recently shown to limit the dinucleoside triphosphate (dNTP) pool and therefore restrict virus DNA replication during the lytic, productive phase of EBV infection (81). The question of whether increased expression of SAMHD1 affects the cellular dNTP pool and contributes to the DRS phenotype during the early, prelatent phase of EBV infection will require future investigations, as dNTPs might also play a role in the very first days of EBV-infected B cells (82).

These additional data suggested that EBV infection does not globally induce cellular genes involved in DNA damage signaling or repair early after infection as reported previously (45), but rather, it selectively regulates genes with dedicated functions in DRS, such as SAMHD1, Chk1, and MRE11A. This interpretation is supported by our experimental findings with the histone variant H2A.X, which is highly phosphorylated at serine 139 on days 3 and 4 (Fig. 9A), when the cells undergo the first round of DNA synthesis and rapid cell divisions, respectively. Primarily, phosphorylation of H2A.X is a sensitive marker for DNA double-strand breaks, which is the canonical function of γ-H2A.X with a central role in DDR (83, 84). Yet, phosphorylation of H2A.X also serves other functions for example in embryonic stem cells, during cellular senescence, and in X chromosome inactivation (see reference 85 for a recent review). In cycling cells, phosphorylation of H2A.X increases as cells progress from G_1_ into the S, G_2_, and M phases, reaching maximal levels at metaphase in the absence of DNA damage (47, 86, 87). H2A.X phosphorylation might have a role in regulating the activation of the mitotic spindle assembly checkpoint (SAC) or in the formation of an intact mitotic checkpoint complex during mitosis (85), documenting that cycling cells show elevated but physiological levels of γ-H2A.X. The DNA damage checkpoint kinase ATR is always active during each S phase, which is due to longer stretches of single-stranded DNA that inherently activate ATR (88) to orchestrate the resolution of stalled replication forks to avoid DNA damage, eventually (49).

We interpret the elevated γ-H2A.X levels on days 3 and 4 p.i. (Fig. 9A) to reflect the cells’ highly proliferative state in the prelatent phase. This interpretation is clearly supported by the ATR inhibitor AZ20, which reduces the elevated γ-H2A.X levels in S- and G_2_/M-phase cells only (Fig. 9D), whereas the ATM inhibitor KU-55933 is not effective and can be even counterproductive (Fig. 9C). Two recent publications are in line with DRS in rapidly cycling B cells in the very early phase of EBV infection (82, 89). Interestingly, the two papers independently show that inhibiting DRS in this early phase of viral infection is counterproductive and does not support the survival of EBV-infected B cells, suggesting that DRS is an epiphenomenon induced by rapid rounds of cellular DNA replication and not genuinely linked to an antiviral response of the host cell.

In contrast to a previous publication (45), EBNA3A and EBNA3C do not seem to control a cellular DDR as indicated by measuring H2A.X phosphorylation (Fig. 9A), negating their roles in a presumed antiviral response of EBV-infected cells. The slight increase in γ-H2A.X detection in cells infected with ΔEBNA3A/C EBV on day 5 and later (Fig. 9A) is in line with a moderately increased proliferation of these B cells (Fig. 8) but is inconsistent with the previous report by Nikitin et al. (45). Several parameters could be the cause of the controversial results, such as virus titers, cell-to-cell variations, B-cell purification methods, or readout techniques, among others. In this context, it seems remarkable that we hardly see any arrested, i.e., noncycling B cells on day 5 p.i. or later in our experiments, suggesting that a nonoptimal multiplicity of infection (MOI of ∼5 in reference 45) has likely contributed to the previous findings. Clearly, multiplicity of infection is a very critical parameter regarding success of viral infection (Fig. 1), but also, initial virus concentrations in the supernatants of the EBV producer cells, i.e., the quality of the virus stock, might be influential. In all the infection experiments reported here (with the exception of those in Fig. 11), we used supernatants with titers of at least 1 × 10^5^ GRU/ml. In our experience, concentration of virus stocks by ultracentrifugation or filtration will lead to the concomitant concentration of cellular debris, subcellular particles, and vesicles, which can be toxic to primary B lymphocytes.

### Programmatic steps in the prelatent phase.

With the exception of B lymphocytes infected with the ΔEBNA2 (5968) EBV mutant, all EBV-infected cells show the same temporal pattern of cellular activation, cell cycle entry, and continuous cell proliferation during EBV’s prelatent phase. This pattern defines clearly discernible phenotypes of the infected cells, as follows: (i) initial growth in cell size with a peak on day 4 p.i. (Fig. 2), (ii) first detection of DNA synthesis exactly on day 3 p.i. (Fig. 3, 8, and 10), (iii) occurrence of the first cell division on day 4 (Fig. 3, 8, and 10), (iv) a phase with very rapid cellular divisions until day 6 p.i. (Fig. 3, 8, and 10) that leads to (v) a decelerated, asynchronously proliferating cell populations eventually. Only B cells infected with EBV mutants negative for LMP2A, EBNA-LP, EBNA1, or EBV’s many miRNAs showed a reduced rate of initial cell divisions during the first 8 days of infection, but the timing of all phases was not altered and extremely reproducible (Fig. 5, 6, 7, and 10). This is surprising and speaks for a coordinated temporal reprograming of the infected cells that is intrinsic and probably depends on the early spike of EBNA2 expression on day 2 p.i. (Fig. 12A). It is attractive to speculate that MYC expression that follows suit is the only driver of this program (90) until other viral factors contribute to and stabilize the activated and reprogrammed cells. Interestingly, upon oncogenic induction of MYC (91), hyperproliferating cells have a short G_1_ phase. Its length is insufficient to correctly position and license replication origins leading to DRS in the subsequent S phase (91). This recent finding is clearly in line with the phenotypes on day 3 and 4 (Fig. 9A and B), supporting our conclusions.

The molecular events that initiate B-cell activation seem to differ from those that lead to activated T cells upon antigen encounter or after CD3 and CD28 stimulation. This is because T lymphocytes react much more rapidly upon activation than B lymphocytes and enter a hyperproliferative phase within 24 h after activation (see references 92 and 93 for a recent review). The reasons for this apparent difference are unknown and probably worth studying.

We took great care to find optimal conditions of B-cell isolation and cultivation and choosing an optimal virus dose, which is a very critical parameter for the survival of EBV-infected B cells (Fig. 1). However, independent of the source of the B lymphocytes (secondary lymphatic tissue or PBMC) or their isolation procedure (FACS sorting or their “untouched” isolation by using magnetic bead-coupled depleting antibodies directed against surface antigens that B cells lack), we always noticed a considerable loss of viable cells during the first 3 days of infection accompanied by a varied but generally high fraction of annexin V binding cells.

Currently, we do not know the origin of this observation. Given our optimal infection conditions, it is very likely that EBV initially infects more than 90% of the B lymphocytes, although the experiment shown in Fig. 1C detected considerably fewer EBNA2-positive cells on day 1 p.i. This technical limitation is obvious when we analyze established lymphoblastoid cell lines by intracellular staining with our EBNA2 specific antibody. Depending on the individual cell sample, this approach detects a fraction of about 60% to 90% EBNA2-positive cells only (data not shown), although all cells are expected to express this essential latent viral gene. EBER hybridization is the common method to detect latently EBV-infected cells, but, in our hands, EBER hybridization was less sensitive than was intracellular EBNA2 staining when the cells were analyzed shortly after infection.

As shown in Fig. 1C, higher MOIs resulted in larger fractions of EBNA2-positive cells on day 1 p.i. and slightly more activated cells than our standard MOI of 0.1 (Fig. 1D), but the initial loss of B lymphocytes is comparable (Fig. 1A and B), and the number of B blasts on day 8 p.i. is reduced at higher MOIs at the start. The initial loss of B lymphocytes is also apparent when the cells are cultivated on CD40L-expressing feeder cells in the presence of IL-4 (Fig. 4). A subpopulation of B lymphocytes that is refractory to EBV or CD40L/IL-4-mediated activation and cell cycle entry would explain this initial failure of B-cell survival. For example, certain B lymphocytes might be incapable of becoming reprogrammed because their epigenetic or metabolic states prevent it. We have no evidence for this assumption but are currently investigating the different possibilities to identify and characterize the obstacles EBV encounters when it infects primary, resting B lymphocytes.

## MATERIALS AND METHODS

### Construction of mutant EBVs.

All modifications of maxi-EBV plasmids described in this study rely on published techniques using homologous recombination in E. coli with linear DNA fragments (94). Following a recent development (95), we constructed a dual-selection cassette consisting of the entire E. coli ribosomal S12 gene (*rpsL*) upstream of the aminoglycoside phosphotransferase gene (*aph*), which is also under the control of the *rpsL* promoter. The expression of *rpsL* results in streptomycin sensitivity at 1 mg/ml streptomycin sulfate in *rpsL*-deficient E. coli strains, whereas *aph* expression results in kanamycin resistance at 40 μg/ml kanamycin sulfate. This dual-selection cassette was cloned onto the pJET1.2 (Fermentas) plasmid to yield the plasmid termed p6012. The entire *rpsL-kana* selection cassette is only 1,348 bp in length and can be amplified with a PCR primer pair (see Text S1 in the supplemental material).

In this study, all recombinant EBVs are based on the maxi-EBV plasmid p2089, which comprises the entire B95.8 EBV genome cloned onto a mini-F-factor plasmid in E. coli (12). For this study, we modified the prokaryotic backbone of this maxi-EBV plasmid in E. coli to encode an artificial open reading frame consisting of the enhanced green fluorescence protein [eGFP], a T2A motif, and the gene encoding resistance against puromycin under the control of the human cytomegalovirus immediate early promoter and enhancer. Puromycin resistance is conferred by the *pac* gene encoding a puromycin *N*-acetyltransferase and replaces the *hph* gene coding for hygromycin B phosphotransferase in p2089. The maxi-EBV plasmid based on p2089, which confers resistance against puromycin, is called p6001 in our plasmid database (Table 1).

The EBV strain B95.8 suffers from a deletion that eliminates multiple miRNA loci, a second lytic origin of DNA replication, *oriLyt*, and additional genes that all field strains of EBV contain. We reconstructed a wild-type like strain based on B95.8 that expresses all viral miRNAs at their physiological levels. We introduced a DNA fragment derived from the EBV strain M-ABA and introduced it into the maxi-EBV plasmid p2089 to repair its deletion. In a second step, we replaced part of the bacterial mini-F factor backbone with the artificial open reading frame encoding both eGFP and *pac* as in p6001 described in the previous paragraph. The resulting maxi-EBV plasmid was very carefully analyzed with numerous restriction enzymes, and DNA sequencing confirmed the two introduced loci and their intact flanking regions. The reconstructed wild-type EBV strain was termed r_wt/B95.8 (6008) (Table 1). It carries the right-handed *oriLyt* and expresses all 25 EBV-encoded pre-miRNAs from their endogenous viral promoters, as well as the LF1, LF2, and LF3 genes that B95.8 EBV lacks.

Subsequently, we replaced the viral miRNA loci with scrambled sequences as described for the previously published EBV ΔmiR (4027) mutant (42) abrogating the expression of all viral miRNAs. Extensive DNA sequencing confirmed all modified loci. The resulting maxi-EBV is called r_ΔmiR (6338) mutant and is listed in Table 1. The complete sequence information of the 25 original and scrambled miRNA loci in the r_ΔmiR (6338) mutant and their secondary structure predictions can be found in Fig. S7.

10.1128/mBio.01723-19.7 FIG S7 Alignments and predicted structures of mutant miRNAs. This multipage figure shows alignments (pages 1 to 5) and predicted secondary structure images (pages 6 to 12) of the 25 pre-miRNAs encoded by EBV field strains (represented by the EBV GenBank accession no. AJ507799) and the corresponding sequences in the mutant EBV strain r_ΔmiR (6338) that carries 25 scrambled miRNA genes. Secondary structures were predicted using the Vienna RNAfold package (107) and are indicated by bracket notations above and below the aligned sequences. Regions that encode mature miRNA sequences or their scrambled counterparts are shown in boldface on a gray background in the alignments or in red in the structure images. The labeling to the left of the aligned sequences denotes the particular miRNAs that correspond to the AJ507799 sequence (shown at the top of each alignment and structure image pair) or are mutated in the r_ΔmiR (6338) mutant EBV strain. The region encoding the mature ebv-miR-BART4* and ebv-miR-BART5* were not scrambled in the r_ΔmiR (6338) mutant, but the mutations of the mature ebv-miR-BART4 and ebv-miR-BART5 sequences efficiently destroy the pre-miRNA hairpin structures (see structure predictions on pages 7 and 8 for these two miR sequences), ablating the expression of ebv-miR-BART4* and ebv-miR-BART5*. Download FIG S7, PDF file, 0.7 MB. Copyright © 2019 Pich et al. 2019 Pich et al. This content is distributed under the terms of the Creative Commons Attribution 4.0 International license.

Based on p2089, its derivative p6001.1, or the newly constructed r_wt/B95.8 (6008) maxi-EBV, we mutated viral loci or deleted them to obtain multiple EBV derivatives as listed in Table 1. In the following paragraphs, we describe the individual experimental steps leading to functional knockouts of EBNA3A, EBNA3C, and the concomitant knockout of both viral genes. We also describe the construction of a pair of two maxi-EBVs, the ΔEBNA-LP (5969) mutant and its wild-type counterpart wt/B95.8 (5750). The construction of the other newly developed maxi-EBVs described in this study (Table 1), such as the ΔEBNA2 (5968), ΔEBER (6431), ΔEBER/ΔmiR (6432), and ΔEBNA1 (6285) mutants, follows the same principles, which are described at the level of DNA sequences in Text S1. More details of all EBV strains, their individual steps of construction, and their annotated DNA sequence files are available upon request. Also available is a step-by-step lab protocol that describes the mutagenesis of EBV genomes in great detail and provides practical hints and hands-on advice.

10.1128/mBio.01723-19.8 TEXT S1 Construction and DNA sequence information of the mutations in eight mutant EBVs. The pages contain the sequence information of the *rpsL-kana* gene cassette cloned into p6012 and detailed DNA sequence information of selected parts of the mutant EBV genomes ΔEBNA1 (6285), ΔEBNA2 (5968), ΔEBNA3A (6077), ΔEBNA3C (6123), ΔEBNA3A/C (6331), ΔEBER (6431), ΔEBNA3A/C (6331), and ΔEBER/ΔmiR (6432), as listed in Table 1. Download Text S1, PDF file, 0.10 MB. Copyright © 2019 Pich et al. 2019 Pich et al. This content is distributed under the terms of the Creative Commons Attribution 4.0 International license.

To establish a mutant EBV with a nonfunctional EBNA3C gene (Table 1), we modified p6001.1 by introducing the *rpsL-kana* cassette of p6012 into the EBNA3c locus. This insertional step was later reverted and replaced by a synthetic DNA fragment with two stop codons in the EBNA3C gene.

In detail, the *rpsL-kana* cassette in p6012 (Text S1) was PCR amplified with two primers, 5′-AGGGATGCTGCCTGCCGGGCTGTCAAGGTGAGTATGCCTCTAACTGGGTTCggcctggtgatgatggcgggatcg and 5′-ACCCTGTTAGGCACGGGAGTTAATGTGCGTAGTGTTGCTGTACGATATCCtcagaagaactcgtcaagaaggcg. The lowercase residues are homologous to sequences in p6012, and the uppercase residues provide the homologous flanks of 50 nucleotides each, which direct the PCR fragment to recombine with the corresponding sequences in p6001.1, located at the 5′ end of EBNA3C from nucleotide coordinates 98704 to 98754 in the upper strand and 98991 to 99040 in the lower strand of the genomic B95.8 sequence, respectively. Recombination was achieved in E. coli strain SW105 (a derivative of the DH10B strain and a kind gift from Neal G. Copeland, Mouse Cancer Genetics Program, National Cancer Institute, Frederick, MC) harboring p6001.1, according to published protocols (94), to yield the maxi-EBV plasmid p6212.1, which was carefully analyzed by restriction enzyme cleavage and sequencing with primers confirming the correct insertion of the *rpsL-kana*-containing PCR fragment.

Next, we used a synthetic DNA fragment (GenScript) of 712 bp in length termed p6122, covering nucleotide coordinates 98505 to 99216 in B95.8. This EBV fragment encompasses parts of the first and second exons of EBNA3C and carries two TGA stop codons, which are in phase with the EBNA3C reading frame terminating translation after amino acid 144 in the first and 209 in the second exon of EBNA3C, corresponding to nucleotide coordinates 98755 to 98757 and 98988 to 98990 in B95.8 DNA, respectively. This DNA fragment released from p6122 with Asp718I cleavage was used to replace the *rpsL-kana* cassette in p6212.1, as described previously (94). In brief, recombination-competent SW105 cells were electroporated with this DNA fragment and subsequently selected on LB plates with chloramphenicol (15 μg/ml) and streptomycin (1 mg/ml) at 32°C. The resulting maxi-EBV plasmid p6123.1, termed ΔEBNA3C (6123), was carefully analyzed by cleavage with several restriction enzymes and partial DNA sequencing to confirm the two point mutations in EBNA3C and the correct insertion of the synthetic DNA fragment encompassing the two stop codons (Text S1).

To inactivate the EBNA3A gene in p6123, we inserted the *rpsL-kana* gene cassette from p6012 into the EBNA3A start codon region in the maxi-EBV plasmid p6123. Two primer pairs, 5′-GGTACAAGGGGGGTGCGGTGTTGGTGAGTCACACTTTTGTTGCAGACAAAggcctggtgatgatggcgggatcg and 5′-AGGACGCCGAATTTTAGGGCGATGCCGAAAAGGTGTCAAGAAATATACAAcgatcccgccatcatcaccaggcc, were used to amplify the *rpsL-kana* cassette (lowercase nucleotide residues) and provide the flanking sequences for homologous recombination (uppercase nucleotide residues) in p6123. The resulting PCR fragment was electroporated into the recombination-competent E. coli SW105 strain carrying the plasmid p6123. DNA recombination deleted 1,348 bp of the first exon and part of the second exon of EBNA3A from nucleotide coordinates 92243 to 93590 in the genomic sequence of B95.8 using the *rpsL-kana* cassette as an insertional mutagen (Text S1).

After selection on chloramphenicol-kanamycin plates, plasmid DNAs were prepared from *rpsL-kana*-positive bacterial clones using the NucleoBond XtraBAC kit and carefully analyzed by restriction enzyme analysis. The resulting EBV plasmid, termed ΔEBNA3A/C (6331), was confirmed by DNA sequencing.

We generated a pair of recombinant EBVs with six copies of EBV’s BamHI-W repeats (Fig. S2). The EBV recombinant wt/B95.8 (5750) is essentially wild type (Table 1), but its derivative ΔEBNA-LP (5969) is incapable of expressing EBNA-LP because each BamHI-W repeat carries a translational stop codon in the W1 exon of EBNA-LP.

To construct the maxi-EBV plasmid pairs wt/B95.8 (5750) and ΔEBNA-LP (5969), we introduced the *galK* gene (94) into wt/B95.8 (2089) deleting a large fragment of EBV DNA from nucleotide coordinates 12001 to 47634 removing the entire cluster of the BamHI-W repeats. The resulting maxi-EBV plasmid was termed p5685.1. Next, we used a single copy of a BamHI-W repeat. Its 5′-BamHI site was changed to a BglII site, and a single nucleotide of its internal BglII site was mutated from 5′-AGATCT to 5′-GGATCT to inactivate it (Fig. S2B). This copy was multimerized to form a 6-mer in pUC18 eliminating all internal BamHI or BglII sites. Both arms of the multimer were then equipped with EBV flanks to generate the plasmid p5537.1. The plasmid was cut with EcoRV and SphI to yield a DNA fragment of 24,896 bp. The left and right EBV flanks of this fragment with sequence homologies of 3,005 bp and 1,716 bp, respectively, mediated a successful DNA recombination with the recipient maxi-EBV plasmid p5685.1 to generate the final wt/B95.8 (5750) maxi-EBV plasmid (Table 1).

Similarly, for the construction of ΔEBNA-LP (5969), we generated a 6-mer of a BamHI-W repeat that is identical to the one in the previous paragraph but carries an additional mutation to inactivate the EBNA-LP gene. Figure S2B illustrates the situation at the sequence level of a single W1 exon. An XbaI site replaced the codons 4 to 7 in the W1 exon introducing a translational stop signal at the fourth codon (in the W1′ exon, the stop codon TAG replaces the second codon) abrogating the translation of any EBNA-LP transcript. The multimer was then equipped with EBV flanks to generate the plasmid p5557.12 in our database. This plasmid was cut with EcoRV and SphI to yield a DNA fragment of 24,896 bp, as described in the paragraph above. DNA recombination with the recipient maxi-EBV plasmid p5685.1 led to the ΔEBNA-LP p5725.1 maxi-EBV plasmid in our database. The backbone of this maxi-EBV plasmid was further altered to remove the *hph* gene but confer resistance against puromycin similar to wt/B95.8 (p6001) to yield the final ΔEBNA-LP (5969) maxi-EBV plasmid (Table 1). The maxi-EBV plasmids wt/B95.8 (5750) and ΔEBNA-LP (5969) were very carefully scrutinized for any sequence alteration with the aid of multiple restriction enzymes and extensive DNA sequencing confirming the genetic compositions of the pair of maxi-EBV plasmids.

For the establishment of producer cell lines, DNAs of all maxi-EBV plasmids listed in Table 1 were further purified by two rounds of CsCl-ethidium bromide density gradient ultracentrifugations prior to their introduction into HEK293 cells.

### Cells and culture.

All cells were maintained in RPMI 1640 medium supplemented with 10% fetal calf serum (FCS), penicillin (100 U/ml), and streptomycin (100 μg/ml). Cells were cultivated at 37°C in a 5% CO_2_ incubator.

### Preparation and quantification of infectious viral stocks.

On the basis of HEK293 cells (obtained from the Leibniz Institut Deutsche Sammlung von Mikroorganismen und Zellkulturen GmbH [DSMZ], Braunschweig, Germany), virus producer cell lines were established after individual transfection of the maxi-EBV plasmid DNAs and subsequent selection with puromycin (500 to 1,000 ng/ml). To obtain virus stocks, clonal producer cells were established on the basis of HEK293 cells, which were transiently transfected with expression plasmids encoding BZLF1 and BALF4 to induce EBV’s lytic cycle. Three days posttransfection, supernatants were harvested and centrifuged at 1,200 rpm for 10 min and 4,800 rpm for 10 min to remove cell debris. The titers of the different virus stocks were quantified, and the concentrations of GFP-transducing virions expressed as green Raji units (GRUs) were determined as described recently in detail (26). A total of 1 × 10^5^ Raji cells (obtained from the Leibniz Institut DSMZ) were incubated with different aliquots of virus stocks at 37°C for 3 days. The percentages of GFP-positive cells were determined by flow cytometry using a BD FACSCanto instrument (BD Bioscience), and the linear regression equation was calculated as described previously (26).

### Quantitation of B95.8 virus stocks.

B95.8 virus supernatants were harvested from B95.8 cells (from the institute’s cell strain collection) and centrifuged to remove cellular debris. A total of 2 × 10^5^ Elijah cells (96) (from the institute’s cell strain collection) were incubated with defined volumes of B95.8 virus stock or a calibrated wt/B95.8 (2089) EBV stock, for which the concentration in GRU per milliliter was known. The cells were incubated at 4°C for 3 h on a roller mixer and washed twice with phosphate-buffered saline (PBS), and the cell pellet was resuspended in 50 μl staining buffer and the anti-glycoprotein 350 (anti-gp350) antibody coupled to Alexa Fluor 647. After incubation at 4°C for 20 min, the cells were washed in staining buffer and resuspended in 300 μl staining buffer, and the MFI values of the stained cells were recorded. A linear regression equation was calculated on the basis of the applied amounts of wt/B95.8 (2089) EBV stock versus MFI, which allowed us to deduce the concentration of the B95.8 virus stock.

### Isolation, separation, and infection of human primary B lymphocytes.

Peripheral B lymphocytes were obtained from 500 ml peripheral blood as buffy coats with a starting volume of about 20 ml. The cells were diluted 10 times with PBS, 0.5 mM acetylsalicylic acid, and 1 mM EDTA to prevent platelet activation. The cells were purified by Ficoll density gradient centrifugation, and the cell-containing interphase was washed several times (1,600 rpm, 1,400 rpm, 1,200 rpm for 10 min each). Remaining erythrocytes were removed with red blood lysis buffer (155 mM NH_4_Cl, 12 mM NaHCO_3_, 0.1 mM EDTA). A total of 2 to 3 × 10^8^ peripheral blood mononuclear cells (PBMCs) were taken up in 2.1 ml staining buffer (PBS, 0.5% bovine serum albumin [BSA], 2 mM EDTA), and 300 μl of anti-human CD61 and CD3 microbeads (Miltenyi Biotec) was added to remove contaminating platelets and T cells, respectively, on 4 LD columns by magnetic sorting, according to the manufacturer’s protocol (Miltenyi Biotec).

Human primary B cells from adenoids were separated from T cells by rosetting with sheep erythrocytes (purchased from Thermo Scientific Oxoid, catalog no. SR0051D) and purified by Ficoll density gradient centrifugation as described in the previous paragraph. Naive B lymphocytes (IgD^+^/IgH^+^, CD38^−^, CD27^−^) were physically sorted on an Aria III instrument (Becton, Dickinson) using the following antibodies: CD38 (eBioscience, catalog no. 25-0389-42), IgD (BD Pharmingen, catalog no. 555778), and CD27 (BD Pharmingen, catalog no. 337169). For virus infection, primary B cells obtained from anonymous donors (see “Ethics Statement,” below) were incubated with virus stocks at a multiplicity of infection of 0.1 GRU for 20 h. After replacement with fresh medium, the infected cells were seeded at an initial density of 1 × 10^6^ cells per ml in 96- or 48-well cluster plates.

B lymphoblasts were generated after plating sorted naive B lymphocytes on irradiated CD40 ligand feeder cells in the presence of IL-4 at 2 ng/ml, as described previously (39).

### Ethics statement.

PBMCs in the form of buffy coats from healthy adult donors were purchased from the Institute for Transfusion Medicine, University of Ulm, Germany. The buffy coats were a side product of standard collection of erythrocytes for medical blood transfusion and were delivered to us in fully anonymized form.

Where indicated, primary naive B lymphocytes were isolated from adenoid samples. They were leftovers from adenoidectomies performed at the Department of Otorhinolaryngology and Head and Neck Surgery, Klinikum der Universität München; these samples also were transferred to our institution in fully anonymized form. The institutional review board (Ethikkommission) of the Klinikum der Universität München, Munich, Germany) approved this procedure (project no. 071-06-075-06).

### Antibodies for flow cytometry analyses or Western blot immunodetection.

For intracellular staining for flow cytometry an Alexa Fluor 647-conjugated mouse monoclonal antibody directed against phosphorylated H2A.X (γ-H2A.X) (clone N1-413) was purchased from BD Bioscience (catalog no. 560447). The rat anti-EBNA2 (1E6) antibody was conjugated with Alexa Fluor 647 and obtained from the in-house Monoclonal Antibody Core Facility.

For Western blotting, the primary mouse monoclonal antibody against Ku70 (clone A-9) was purchased from Santa Cruz Biotechnology, and the rabbit monoclonal antibody specific for p21 (clone EPR3993) was purchased from Abcam. Polyclonal rabbit antibodies directed against p53 (no. 9282) and Rad51 were purchased from Cell Signaling and Santa Cruz Biotechnology, respectively. Rat antibodies specific for EBNA2 (R3 and 1E6), EBNA1 (1H4), and EBNA3A (clone E3AN 4A5-11111) proteins and mouse antibodies specific for EBNA-LP (JF186), MYC (9E10), and EBNA3C proteins (clone E3A10-P2-583) obtained from the in-house Monoclonal Antibody Core Facility were used.

### Intracellular staining of γ-H2A.X for flow cytometry.

The infected cells were treated with 85 μM etoposide for 1 h or left untreated before harvesting at the indicated time points p.i. Intracellular flow cytometry stainings were performed with fixed cells using the Fix & Perm kit (Thermo Fisher Scientific). Briefly, 5 × 10^5^ cells were washed and suspended in 100 μl PBS. Cells were fixed by adding 100 μl Fix & Perm medium A for 15 min in the dark at room temperature. The fixed cells were washed with 1 ml staining buffer (PBS, 0.5% BSA, 2 mM EDTA) and the pellet resuspended in 1 ml freezing medium (90% FCS, 10% dimethyl sulfoxide [DMSO]) to be stored at –80°C. After sample collection was completed, the cells were thawed, washed again, and centrifuged, and the pellets were resuspended and permeabilized in 100 μl Fix & Perm medium B. During the permeabilization step, anti-γ-H2A.X staining was performed for 20 min at room temperature in the dark. γ-H2A.X specific mouse monoclonal antibody-conjugated Alexa Fluor 647 was diluted 1:50. After another washing step with 1 ml staining buffer, the samples were measured with a BD LSRFortessa or FACSCanto cell analyzer (Becton, Dickinson).

### Intracellular staining of EBNA2 for flow cytometry.

The infected cells were harvested at the given time points after infection, pelleted by centrifugation, and resuspended in 100 μl PBS. Cells were fixed by adding 100 μl Fix & Perm medium A (Life Technologies) for 15 min in the dark at room temperature. The fixed cells were washed with 1 ml staining buffer (PBS, 0.5% BSA, 2 mM EDTA), pelleted, and resuspended in 1 ml freezing medium (90% FCS, 10% DMSO) to be stored at –80°C. After sample collection, the cells were thawed and washed in 1 ml staining buffer. The pellet was resuspended in 100 μl Fix & Perm medium B (Life Technologies), and 1.7 μl of a 1:10 dilution of the anti-EBNA2 monoclonal antibody 1E6 coupled with Alexa Fluor 647 (1:600 final) was added. The cells were incubated at room temperature for 20 min, washed, and resuspended in staining buffer for flow cytometry analysis.

### BrdU incorporation and cell cycle analysis.

Metabolic BrdU labeling and detection of newly synthesized DNA were performed by adding 10 μM BrdU (APC BrdU flow kit, catalog no. 557892; BD Pharmingen) for 1 h prior to harvest. The cells were washed in staining butter (PBS, 0.5% BSA, 2 mM EDTA), and the pellets were resuspended and fixed in 100 μl Cytofix/Cytoperm buffer (APC BrdU flow kit) and incubated on ice for 20 min. The cells were washed in staining buffer, pelleted, and resuspended in 1 ml freezing medium (90% FCS, 10% DMSO) to be stored at –80°C. After sample collection, the cells were thawed and washed in 1 ml staining buffer. The pellet was resuspended in 100 μl Cytofix/Cytoperm buffer (APC BrdU flow kit) incubated on ice for 5 min, washed in 1 ml Perm & Wash buffer (APC BrdU flow kit), and further processed according to the manufacturer’s instructions. Cellular DNA was counterstained with 7-amino-actinomycin D (7-AAD; BioLegend), and the cells were analyzed on a BD LSRFortessa instrument.

### EdU incorporation and inhibitors of DDR and DRS.

Metabolic EdU labeling and detection of newly synthesized DNA were performed with the Click-iT EdU flow cytometry cell proliferation assay by Thermo Fisher Scientific and Alexa Fluor 488 azide. The cells were metabolically labeled with 10 μM EdU for 1 h and analyzed following the manufacturer’s instructions. KU-55933 (SML 1109) and AZ20 (SML 1328) were purchased from Sigma-Aldrich and used at final concentrations of 10 μM and 2 μM, respectively.

### Cell size and TMRE staining.

Primary naive B lymphocytes were infected with wt/B95.8 (2089) EBV with an MOI of 0.1 and analyzed daily for their cell diameter. Briefly, we purified intact cells by Ficoll density gradient centrifugation, washed the cells in PBS, and recorded microscopic images of the cell samples using a Tali image-based cytometer (Thermo Fisher). We interpreted the images using the ImageJ software package and identified cellular objects with circularity of >0.75 and aspect ratio of ≦1.3 as intact cells. The individual diameters of each cell were calculated based on images obtained with calibration beads of a radius of 1.9 mm on average (Tali calibration beads; Thermo Fisher). The mean and standard deviation of the cells’ diameters were determined accordingly (GraphPad Prism software, version 7) and the volumes of cells were calculated assuming a spherical shape. The incorporation of TMRE has been described recently (27).

### Protein lysates and Western blot immunodetection.

The infected cells were harvested at the indicated times postinfection. Cells were washed with phosphate-buffered saline (PBS) and lysed with lysis buffer (0.02% sodium dodecyl sulfate [SDS], 0.5% Triton X-100, 300 mM NaCl, 20 mM Tris-HCl [pH 7.6], 1 mM EDTA, 1 mM dithiothreitol) for 10 min on ice. Samples were centrifuged at 10,000 × *g* for 1 min at 4°C. Samples were adjusted to 5 × 10^7^ cells/ml lysis buffer.

Six percent polyacrylamide gels were used for EBNA3A and EBNA3C, and for other proteins, 12% polyacrylamide gels were used. Ten microliters of protein samples (lysate from 5 × 10^5^ cells) was loaded per lane. Protein samples were separated by SDS-PAGE and subsequently transferred onto 0.45 μM nitrocellulose membranes (Bio-Rad) for 60 min at 100 V in a Mini-Protean tetra cell (Bio-Rad). For EBNA3A and EBNA3C detection, proteins were transferred for 90 min. Membranes were blocked with 5% milk in PBS containing 0.05% Tween 20 (PBS-T) for 1 h at room temperature. Membranes were probed as indicated; the anti-Ku 70 mouse monoclonal antibody was diluted 1:1,000 and incubated for 1 h at room temperature, the anti-p21 rabbit monoclonal antibody was diluted 1:5,000 and incubated at 4°C overnight, the anti-p53 rabbit polyclonal antibody was diluted 1:1,000 and incubated at 4°C overnight, the anti-Rad51 rabbit polyclonal antibody was diluted 1:500 and incubated at 4°C overnight, and the anti-EBNA2 rat polyclonal antibody was diluted 1:200 and incubated at 4°C overnight. Horseradish peroxidase (HRP)-conjugated species-specific goat secondary antibodies were used at a 1:5,000 dilution in PBS-T, and the membranes were incubated for 1 h at room temperature. The signals were visualized by exposure to X-ray film.
