# Supplementary figures and images for: First Days in the Life of Naive Human B Lymphocytes Infected with Epstein-Barr Virus
Source: mBio. 2019 Sep 17;10(5):e01723-19. doi: 10.1128/mBio.01723-19 (PMC6751056; doi:10.1128/mBio.01723-19)

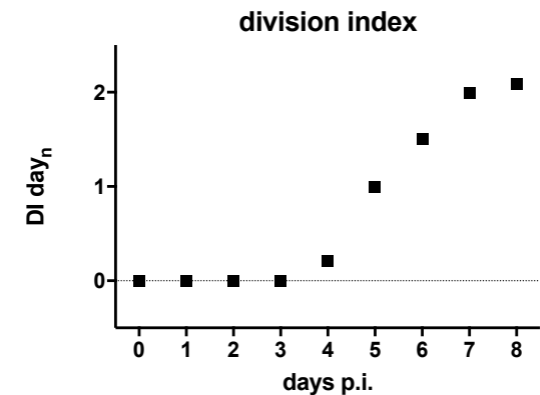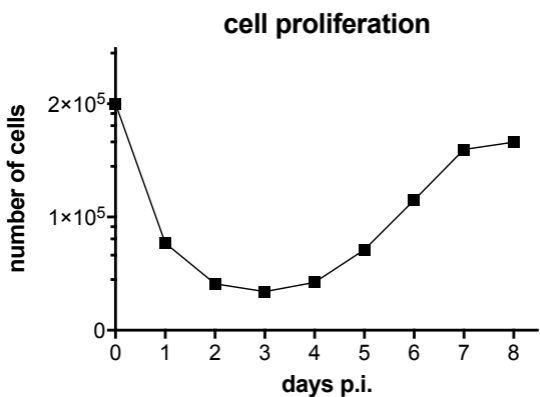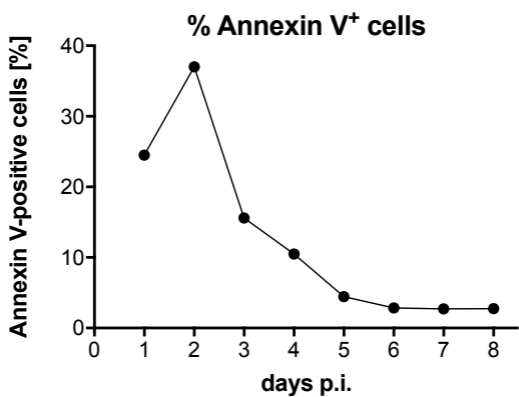

Fig. S1

Supplement: FIG S1 [file mBio.01723-19-sf001.pdf]

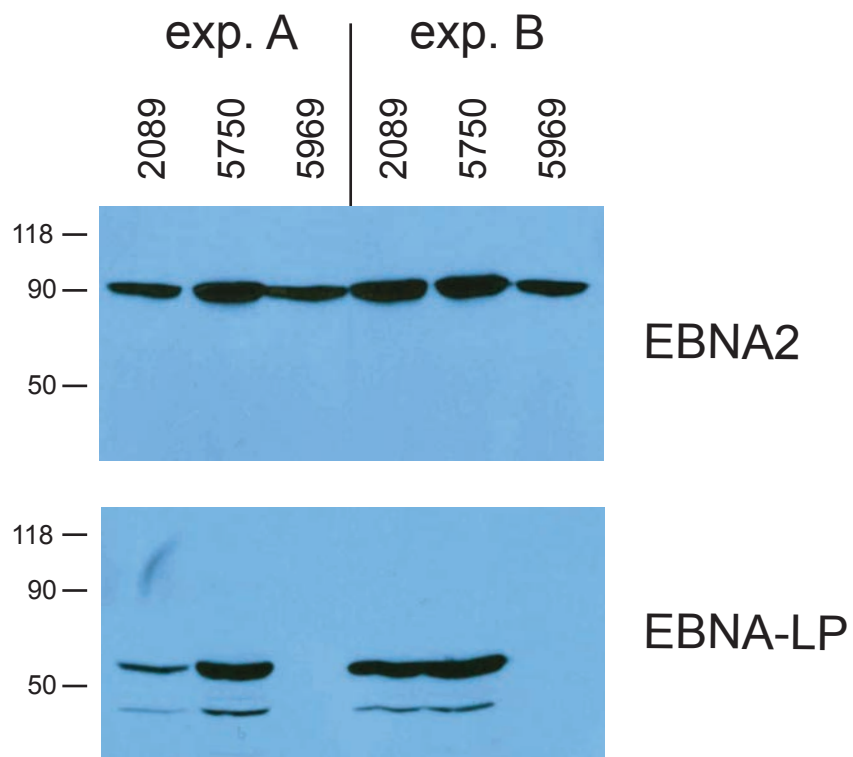

Fig. S3

Supplement: FIG S3 [file mBio.01723-19-sf003.pdf]

## cell proliferation

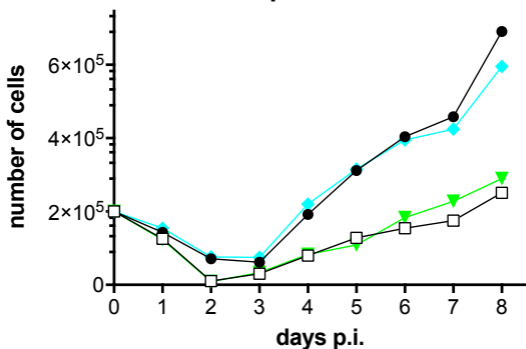

## % apoptosis

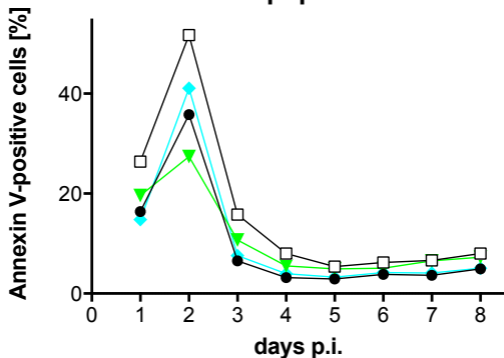

- r\_wt/B95-8 (6008)
- ▼ r\_ΔmiR (6338)
- ΔEBER, ΔmiR (6432)
- ◆ ΔEBER (6431)

Fig. S4

Supplement: FIG S4 [file mBio.01723-19-sf004.pdf]

wt/B95.8 (2089)

$\Delta$ EBNA3A/C (6331)

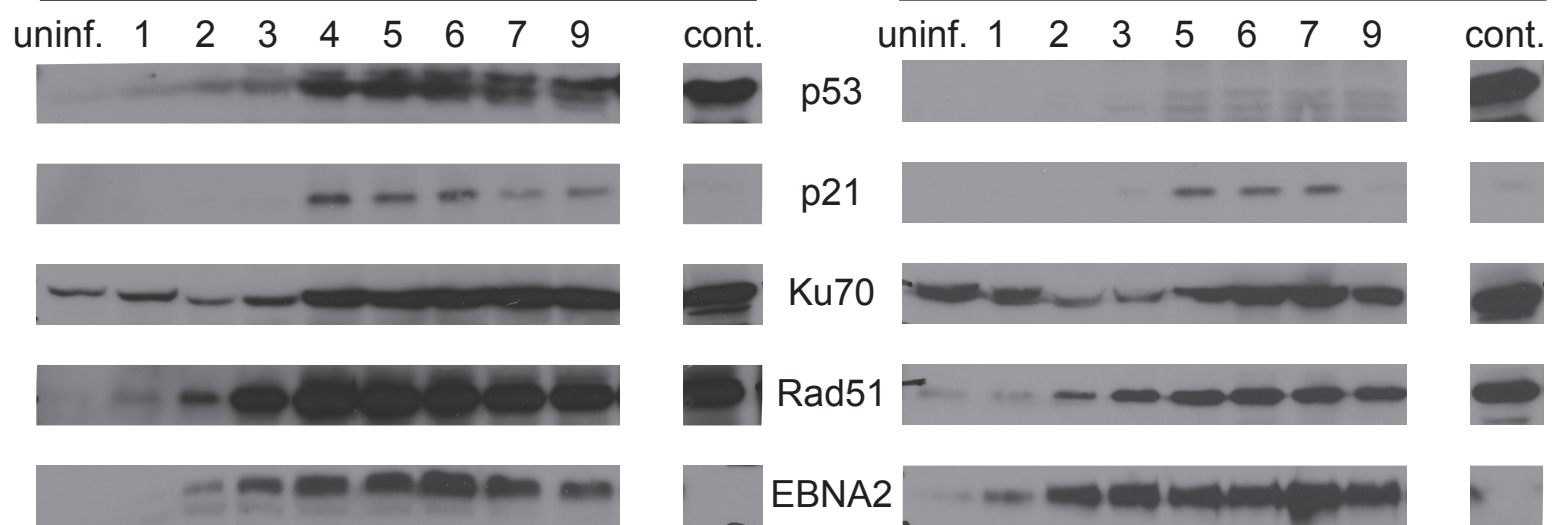

Fig. S5

Supplement: FIG S5 [file mBio.01723-19-sf005.pdf]

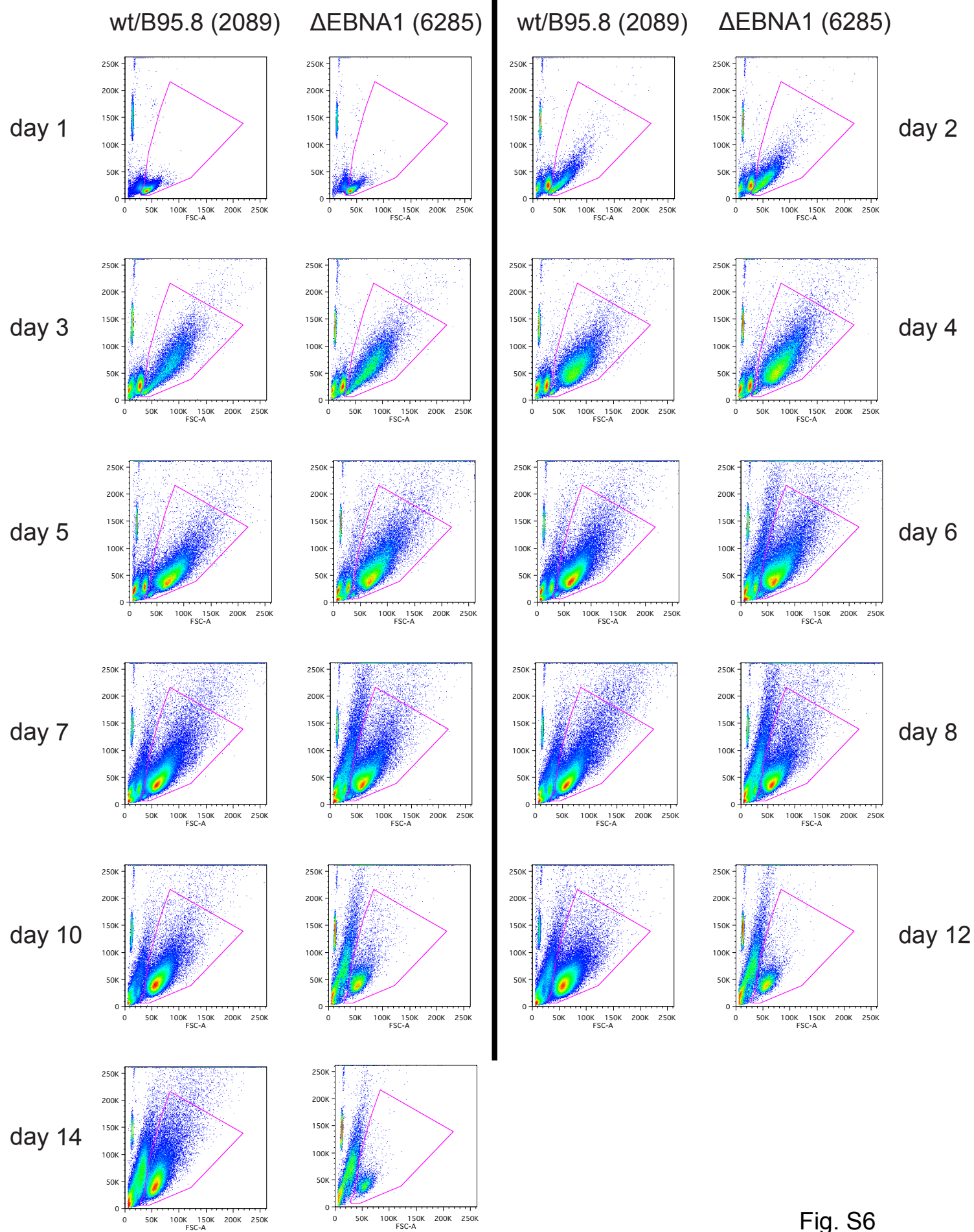

Fig. S6

Supplement: FIG S6 [file mBio.01723-19-sf006.pdf]
