# Supplementary material for: First Days in the Life of Naive Human B Lymphocytes Infected with Epstein-Barr Virus
Source: mBio. 2019 Sep 17;10(5):e01723-19. doi: 10.1128/mBio.01723-19 (PMC6751056; doi:10.1128/mBio.01723-19)
Supplement: FIG S2 [file mBio.01723-19-sf002.pdf]

A

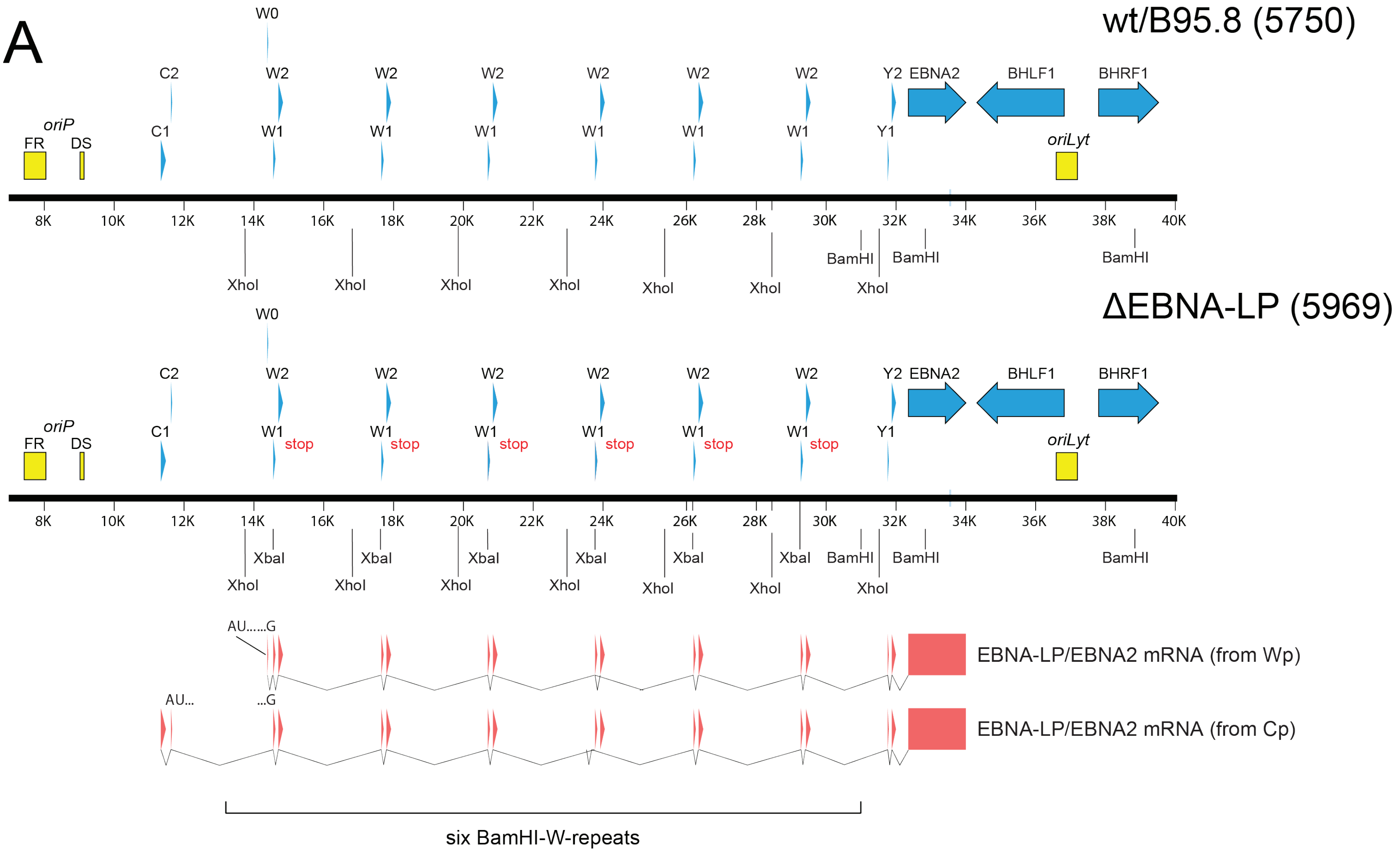

B

C2/BamHI-W-repeat array in wt/B95.8 (2089)

C2------BamHI------BglII-------W0---W1/W1'---W2------BamHI---

GGATCC AGATCT GGATCC
CCTAGG TCTAGA CCTAGG

C2/BamHI-W-repeat array in wt/B95.8 (5750) and ΔEBNA-LP (5969)

C2-----(BamHI)----(BglII)------W0---W1/W1'---W2-----(BamHI)--

GGATCT GGATCT GGATCT
CCTAGA CCTAGA CCTAGA

W1 exon in wt/B95.8 (5750)

CCT AGG GGA GAC CGA AGT GAA GGC CCT GGA CCA ACC CGG CCC GGG CCC CCC GGT ATC GGG CCA GAG
GGA TCC CCT CTG GCT TCA CTT CCG GGA CCT GGT TGG GCC GGG CCC GGG GGG CCA TAG CCC GGT CTC
P R G D R S E G P G P T R P G P P G I G P E

W1 exon in ΔEBNA-LP (5969)

CCT AGG GGA TAG TCT AGA CTA GGC CCT GGA CCA ACC CGG CCC GGG CCC CCC GGT ATC GGG CCA GAG
GGA TCC CCT ATC AGA TCT GAT CCG GGA CCT GGT TGG GCC GGG CCC GGG GGG CCA TAG CCC GGT CTC
P R G *

Fig. S2
