# Supplementary material for: First Days in the Life of Naive Human B Lymphocytes Infected with Epstein-Barr Virus
Source: mBio. 2019 Sep 17;10(5):e01723-19. doi: 10.1128/mBio.01723-19 (PMC6751056; doi:10.1128/mBio.01723-19)
Supplement: FIG S7 [file mBio.01723-19-sf007.pdf]

```
ebv-miR-BHRF1-1
                             ebv-miR-BHRF1-1
                (((.((((.(((((((..((((.(((((.((((.............)))).))))).))))..)))))))....)))))))
AJ507799        GGCUCCUUAUUAACCUGAUCAGCCCCGGAGUUGCCUGUUUCAUCACUAACCCCGGGCCUGAAGAGGUUGACAAGAAGGGUC
                |||||||||| |   | | | |        |||||||||||||||||||||||||||||||||||||||||||||||||||
r_ΔmiR(6338)    GGCUCCUUAUGAUAAUAACCCGGGUGCCCCUUGCCUGUUUCAUCACUAACCCCGGGCCUGAAGAGGUUGACAAGAAGGGUC
                (((.((((.((.......((((((.((.....))..(((........))))))))))(((....)))....))..)))))))
```

```
ebv-miR-BHRF1-2
                               ebv-miR-BHRF1-2*                    ebv-miR-BHRF1-2
                ((((..(((((((((.(((((((((((.((((((.............)))))).))))))))))).)))))))))..))))
AJ507799        GGCCCCCACUUUUAAAUUCUGUUGCAGCAGAUAGCUGAUACCCAAUGUUAUCUUUUGCGGCAGAAAUUGAAAGUGCUGGCC
                |||||||||||||   |          || |    ||||||||||||| |   ||   |   |  |||  |||||||||||
r_ΔmiR(6338)    GGCCCCCACUUUUCGGUAGGUGAUAUCCAAACUAUUGAUACCCAAUGUGAAACUUCUGGAUCGUGAUUAUAAGUGCUGGCC
                ((((..(((((.(((((((............)))))))...(((............)))...........)))))..))))
```

```
ebv-miR-BHRF1-3
                               ebv-miR-BHRF1-3
                ((((((.((((.((((((..((((((.((((((...(((.........)))))))))))))))..)))))).)))).))).)))
AJ507799        CCUGUGGUGUUCUAACGGGAAGUGUGUAAGCACACACGUAAUUUGCAAGCGGUGCUUCACGCUCUUCGUUAAAAUAACACAAGG
                ||||||||||||| | |  |||       | |  ||||||||||||||||||||||||||||||||||||||||||||||||||
r_ΔmiR(6338)    CCUGUGGUGUUCUGAAGAUAAGGUCCACGGAAUGCACGUAAUUUGCAAGCGGUGCUUCACGCUCUUCGUUAAAAUAACACAAGG
                ((((((((((((((..(((...)))..))))))))............(((((.((.....))...))))).......))).)))
```

```
ebv-miR-BART3
                             ebv-miR-BART3*                       ebv-miR-BART3
                (((((((((..((((.(((((((((.((((((..............)))))).)))))).))).))))..)))))))))
AJ507799        CCUUUGGUGGAACCUAGUGUUAGUGUUGUGCUGUAAAUAAGUGUCCAGCGCACCACUAGUCACCAGGUGUCACCGGAGG
                ||||||||||| |||||| |  ||  |||  ||||||||||||||||| |   |       | | |  | |||||||||
r_ΔmiR(6338)    CCUUUGGUGGAGCCUAGUUUCGGUUGUGUAAUGUAAAUAAGUGUCCAGUGUGUCCACCACAAACUGCCGGCACCGGAGG
                (((((((((.((((.......))))....................((((.(((.....))).))))....)))))))))
```

```
ebv-miR-BART4
                               ebv-miR-BART4                          ebv-miR-BART4*
                ((...((((((((.(((((.(((((((((((((((..............))))))))).)))).)).))))).))))))))..))
AJ507799        UAACAUUUGGUGGGACCUGAUGCUGCUGGUGUGCUGUAAAUAAGUGCCUAGCACAUCACGUAGGCACCAGGUGUCACCAGGGCUA
                ||||||||||||| |     |  ||     ||  ||||||||||||||||||||||||||||||||||||||||||||||||||||
r_ΔmiR(6338)    UAACAUUUGGUGGCAGAGCCUUUUGGGUCGGUCGUGUAAAUAAGUGCCUAGCACAUCACGUAGGCACCAGGUGUCACCAGGGCUA
                .....((((((((((((.((....)).))..............((((((.((.......))))))))....))))))))))....
```

```
ebv-miR-BART1
                          ebv-miR-BART1-5p                    ebv-miR-BART1-3p
               ((((.((.(((...(((((((((((..((((((((.........))))))))))).)))))))...))).))))))
AJ507799       CCCGUGGGGGGUCUUAGUGGAAGUGACGUGCUGUGAAUACAGGUCCAUAGCACCGCUAUCCACUAUGUCUCGCCCGGG
               ||||||||||||    |     | |  | |||    ||||||||||||| |       |  ||        |||||||||
r_ΔmiR(6338)   CCCGUGGGGGGUGGGACCUUAUGGUAUGUGGAUCGAAUACAGGUCCACAAUUAGAUUCACCCUCCCUGGUCGCCCGGG
               .(((.(((((((((.(((....))).((((((((.......)))))))).......))))))))).)))........
```

```
ebv-miR-BART15
                                                       ebv-miR-BART15
               .(((...(((..(((((((((.((((((.(((..((((.....)))))))))))))).)))))))))....))).))).
AJ507799       UGUGCCGCUUGGAGGGAAACAUGACCACCUGAAGUCUGUUAACCAGGUCAGUGGUUUUGUUUCCUUGAUAGAGACACA
               ||||||||||||||||||||||||||||||||||||||||||||||      | ||||||||         ||||||||||
r_ΔmiR(6338)   UGUGCCGCUUGGAGGGAAACAUGACCACCUGAAGUCUGUUAACCAGUCGCUUUGUUUUGUUGUGACAGUAGAGACACA
               .(((...(((...((..((((.(((.........)))))))..)).(((((...........)))))....))).))).
```

```
ebv-miR-BART5
                        ebv-miR-BART5                            ebv-miR-BART5*
               (((.(((....((((((((..(((.((((((((................)))))))).)))))))))))...))).)))
AJ507799       GUGGCACCUCAAGGUGAAUAUAGCUGCCCAUCGACGUAUCGCUGGAAACCGGUGGGCCGCUGUUCACCUAAAGUGACGC
               |||||||||||||||||||||||||||||||||||||||||||||||||||||||||||||||    |        |  |  |
r_ΔmiR(6338)   GUGGCACCUCAAGGUGAAUAUAGCUGCCCAUCGACGUAUCGCUGGAAACCGGUGGGCCAGGGCCUGGCCGCACGGGCCA
               ....((((....)))).........((((((((..((.((....)).)))))))))).((((.....))))........
```

```
ebv-miR-BART16
                              ebv-miR-BART16
               (((.(((((.((...((((((..(((((.(((.(((((...........))))).))))))))))))))...)).))))).)))
AJ507799       CAGGUGUGGAAUUUAGAUAGAGUGGGUGUGUGCUCUUGUUUAAUUACACCAAGAUCACCACCCUCUAUCCAUAUCCCACAAUUG
               ||||||||||||      |     |     ||||   |||||||||||||||||||||||||||||||||||||||||||||||||
r_ΔmiR(6338)   CAGGUGUGGAAUCGGAUUGAUACGAUGUUGUGGGUUUGUUUAAUUACACCAAGAUCACCACCCUCUAUCCAUAUCCCACAAUUG
               (((.(((((.((((........))))...((((.((((...........))))....))))..............))))).)))
```

```
ebv-miR-BART17
                     ebv-miR-BART17-5p                       ebv-miR-BART17-3p
               (((...(((((((.(((((((((((((((((((............))))))))))))).)))))).))))).))..)))
AJ507799       GUGGCACCCUAAGAGGACGCAGGCAUACAAGGUUAUUACCCAGUCCUUGUAUGCCUGGUGUCCCCUUAGUGGGACGC
               |||||||||     | |             | |||||||||||||||| ||   || |         |      |||||||
r_ΔmiR(6338)   GUGGCACCCCCGAGGAAAAACUAACGGUAGGGUUAUUACCCAGUCCUCGUCAGCGUUCGUAUUUCGGUUCGGGACGC
               ..(((.....((((((...((.....)).((((....))))..))))))...((((((...............))))))
```

```
ebv-miR-BART6
                             ebv-miR-BART6-5p                       ebv-miR-BART6-3p
               ((((....(((((((((((((((((.((((...((.((((......)))).))..)))).)))))))))))))))))...))))
AJ507799       CUUGUUGGUACUUUAAGGUUGGUCCAAUCCAUAGGCUUUUUUUGUGAAAACCCGGGGAUCGGACUAGCCUUAGAGUAACUCAAG
               ||||||||||||||  |  | |    |        |||||||||||||||||  |    |              ||||||||||
r_ΔmiR(6338)   CUUGUUGGUACUUUCUGAAUCGCGAGAGUUUGCAACUUUUUUUGUGAAAACCAAGACCGCCACUGGUGUGAGUGGUAACUCAAG
               ((((.((((.(((((........)))))((..(((......)))..)).))))..((((((((....))).)))))....))))
```

```
ebv-miR-BART21
                              ebv-miR-BART21-5p                 ebv-miR-BART21-3p
               ((.(((((((((((..((((((((...(((((((.(((........))).)))))))...))))))))..))))))).)))).))
AJ507799       GUAUGGGCUGGGUAUUCACUAGUGAAGGCAACUAACACAGUUAGACGUGCUAGUUGUGCCCACUGGUGUUUAUCCGGUCCCAAAU
               ||||||||||||     |    ||||  | ||  |   ||||||||    |       |   |   |  |   | ||||||||||
r_ΔmiR(6338)   GUAUGGGCUGGGGCCCAAGAGAUGAAAUCUACAUAUCUAGUUAGACUGCUUUUGGUGACGUUCCCAUAGUCUGCGGGUCCCAAAU
               .....(((((((((((.(((.(((.......))).)))...(((((((....((....))......))))))).))))))))...
```

```
ebv-miR-BART18
                             ebv-miR-BART18-5p                  ebv-miR-BART18-3p
               ((((((..((((...(((((((.((((((.((((((.((....)).)))))).)))))).)))))))....))))..))))))
AJ507799       CGGGUGUCCUGGCUCAAGUUCGCACUUCCUAUACAGUGUUAAAGCCUUGUAUCGGAAGUUUGGGCUUCGUCCCAGUGUACUCG
               ||||||||||||||| | |||       ||     ||||||||||||||     | ||     | | |   ||||||||||||
r_ΔmiR(6338)   CGGGUGUCCUGGCUCCAAUUCCACUCAACUGGUAUGUGUUAAAGCCUUGGUGUAGCAGCGGUUGUUACUCUCCAGUGUACUCG
               ((..((((..((((..(((.(((......))).....)))..)))).(((.(((((((...)))))))...)))........)
```

```
ebv-miR-BART7
                                ebv-miR-BART7*                      ebv-miR-BART7
               ((((((..(((.((((((((((.((((((((..((............))..)))))))).)).)))))))).)))..)))).))
AJ507799       CCAGUGUCCUGAUCCUGGACCUUGACUAUGAAACAAUUCUAAAAAAAUGCAUCAUAGUCCAGUGUCCAGGGACAGUGCACUCGG
               ||||||||||||| |  ||      |     ||| ||||||||||||||   |   ||     |   |  ||||||||||||||
r_ΔmiR(6338)   CCAGUGUCCUGAUUCGAGAUUGACGCAUCACAACUAUUCUAAAAAAAUGGCCCUAUGUUAGCCGGAAACUGACAGUGCACUCGG
               .....))))......((((((..((...(((..(((((.......))))).....((((((.......)))))))))....)).
```

```
ebv-miR-BART8
                                ebv-miR-BART8                       ebv-miR-BART8*
               (((((.(((((.((((((..((.((((((((((((...........)))).)))))))).))..)))))).))))).)))))
AJ507799       UGGGUUCACUGAUUACGGUUUCCUAGAUUGUACAGAUGAACUAGAACUGUCACAAUCUAUGGGGUCGUAGACAGUGUGCUUA
               |||||||||||||   |      |  |        ||||||||||||||    | ||| | | |    |  |||||||||||
r_ΔmiR(6338)   UGGGUUCACUGAUCUGGCAGCUUUUUACAAGUUGAAUGAACUAGAACUGAUGUAGUCUUUCGAGGGCAAACCAGUGUGCUUA
               (((((.(((((.(((((((((((.....))))))......))))).........((((.....))))....))))).)))))
```

```
ebv-miR-BART9
                             ebv-miR-BART9*                       ebv-miR-BART9
              (((.((((((..(((.((((((((((.((...((((..........))))..)).)))).)))))).)))..))))))...)))
AJ507799      AGCUGUUGUUUGUACUGGACCCUGAAUUGGAAACAGUAACUUGGAUUCUGUAACACUUCAUGGGUCCCGUAGUGACAACUAUGCU
              |||||||||||||  ||      |          ||||||||||||||||      || | |       |   ||||||||||||
r_ΔmiR(6338)  AGCUGUUGUUUGUCUUGAGGAUAGCCAACAGCUAAGUAACUUGGAUUCUGCCGACUUUGACGCUGUGAAUUCAGACAACUAUGCU
              .(((((((.(((((.....))))).))))))))........(((......)))........))..)).((((((.((....))))
```

```
ebv-miR-BART22
                                                                ebv-miR-BART22
              ((((..(((((((.(((((((((.(((..(((((((.........)))).)))..))).))))))))).))))))).))))
AJ507799      GCGGUUGUCACAGGUGCUAGACCCUGGAGUUGAACCAGUACCACUCGGUUACAAAGUCAUGGUCUAGUAGUUGUGACCCUGC
              ||||||||||||||||||||||||||||||||||||||||||||||            |   | | |||  | |  ||||||
r_ΔmiR(6338)  GCGGUUGUCACAGGUGCUAGACCCUGGAGUUGAACCAGUACCACUCAAGCUGUCGUGGAGAUUAUGGUACAUAUUGCCCUGC
              ..((((.((.((((........))))))....)))).(((((((((..((....)).)))....))))))...)))))))...
```

```
ebv-miR-BART10
                             ebv-miR-BART10*                    ebv-miR-BART10
              ((((((..(((.(((((.((((.(((((.((((((...........)))))).))))).)))).))))).)))..))))))
AJ507799      GGAGUGUCCCGGGGCCACCUCUUUGGUUCUGUACAUAUUUUGUUAUUGUACAUAACCAUGGAGUUGGCUGUGGUGCACUCC
              ||||||||||||||||||||||||||||||||||||||||||||||            |   | | |||  | |  ||||||
r_ΔmiR(6338)  GCGGUUGUCACAGGUGCUAGACCCUGGAGUUGAACCAGUACCACUCAAGCUGUCGUGGAGAUUAUGGUACAUAUUGCCCUGC
              ..((((.((.((((........))))))....)))).(((((((((..((....)).)))....))))))...)))))))...
```

```
ebv-miR-BART11
                             ebv-miR-BART11-5p                   ebv-miR-BART11-3p
              ((((.((((((((.(((.((((((((((.((..(((((.......)))))...)).)))))))))).))).))))))))...))))
AJ507799      GGCUUCUGUUGGGUCAGACAGUUUGGUGCGCUAGUUGUGUGCUUAGCAGCAACGCACACCAGGCUGACUGCCUUAGCAGUGUGGCC
              |||||||||||||         |        | | | |||||||||||||||| | |        | |  | ||||||||||||||
r_ΔmiR(6338)  GGCUUCUGUUGGGCGUUUGGAGUGUCGUAAAUCGGUCUGUGCUUAGCAGCAACCCGCUGUGCCAAGCCGACAUUAGCAGUGUGGCC
              ((((.((((((((((.......))))).....((((..((.((...(((((.....)))))))))..))))...)))))))...))))
```

```
ebv-miR-BART12
                                                                ebv-miR-BART12
              ((((((.(((((.(((((((...((((((.((((((.........)))))).)))))))...)))).))).))))).))).)))
AJ507799      CUGGUGACCUAACACCCGCCCAUCACCACCGGACAGAUUCUGAACUUGUCCUGUGGUGUUUGGUGUGGUUUUGGGGUACGCAG
              ||||||||||||||||||||||||||||||||||||||||||||||||  | | ||  |||  |||| |||||||||||||||
r_ΔmiR(6338)  CUGGUGACCUAACACCCGCCCAUCACCACCGGACAGAUUCUGAACUUGCGCGGGGGGUUUUUUUGUGUUUUUGGGGUACGCAG
              (.((((......)))).))..........((((.....))))....)))))))).((.(((((((((.........))))))
```

```
ebv-miR-BART19
                              ebv-miR-BART19-5p                    ebv-miR-BART19-3p
               (((.(((((((...(((((((.(((((((.((((((............)))))).)))))))..)))))))...))))))).)))
AJ507799       CCGUGUCCUGACAACAUUCCCCGCAAACAUGACAUGGGUUAAUUUAAACAUGUUUUGUUUGCUUGGGAAUGCUCUUAGGGCCUGG
               |||||||||||||     ||    | |||   | | ||||||||||||||||||| ||  | ||  |   |||||||||||||||
r_ΔmiR(6338)   CCGUGUCCUGACACUCGACCAAAGAGACACCUCUUAGGUUAAUUUAAACAUGUUUAGUGCGGUUUUGUUAGCUCUUAGGGCCUGG
               (((.(((((((..........(((((....))))).(((((((..((((.(((.....))))))).))))))).))))))).)))
```

```
ebv-miR-BART20
                           ebv-miR-BART20-5p                  ebv-miR-BART20-3p
               ((((((.((.(((((((((.(((((((((.((............)).)))))))))..))))))))).)).))).)))
AJ507799       AGGGCCUAUUGUAGCAGGCAUGUCUUCAUUCCUGCGUACCGAAUGGCAUGAAGGCACAGCCUGUUACCAUUGGCACCU
               |||||||||||   |          ||   | |||||||||||||| | |  | |    |      | ||||||||||
r_ΔmiR(6338)   AGGGCCUAUUGCUACUCUGUCAAGAUCUGGCUUGCGUACCGAAUGGUAGGCCGCCGGUACAACAAUCUAUUGGCACCU
               ((((((....((((.(((....)))..))))....((((((..(((....))).))))))...........))).)))
```

```
ebv-miR-BART13
                             ebv-miR-BART13*                        ebv-miR-BART13
               (((((.(((.((.(((.(((.((((...((((((............))))))....))))))).))).)).))).)))))
AJ507799       GGCACCUCGAUAACCGGCUCGUGGCUCGUACAGACGAUUGUUUGGCUCUGUAACUUGCCAGGGACGGCUGACGAUGUGUU
               |||||||||||     |  | |    |    |||||||||||||||||        |       |||   ||||||||||
r_ΔmiR(6338)   GGCACCUCGAUUCGGCGGCCAUUAACCCGGUAGACGAUUGUUUGGCUCGCACGUCAGUUCAUAGCGGGGUACGAUGUGUU
               (((((.(((((((.(((((((......((.....))......)))).)))......((.....)).)))).))).)))))
```

```
ebv-miR-BART14
                               ebv-miR-BART14*                    ebv-miR-BART14
               ((((.(((.((((..(((((((((((..(((((((..........)))))))...))))).))))))..))))...)))..))))
AJ507799       CAGGGGUGGCCGGUACCCUACGCUGCCGAUUUACAUAAUAUAAAUUGUAAAUGCUGCAGUAGUAGGGAUCUGGACGCGCGACCUG
               |||||||||||||                |     ||||||||||||    |  ||  | | |    | ||||||||||||||||
r_ΔmiR(6338)   CAGGGGUGGCCGGCCUAUACUCGCCGAACUGCUAUUAAUAUAAAUUGGGGUUCGUGAUGGAAUCUAAAACUGGACGCGCGACCUG
               (((.((((((((((........))))....))))))...........((((((......))))))....))))))..((.....)
```

```
ebv-miR-BART2
                             ebv-miR-BART2-5p                        ebv-miR-BART2-3p
               (((((..(((..((((((((.((.(((((((((((..........))))))).))))..)).))))))))..)))..)))))
AJ507799       GGACUUCCAGACUAUUUUCUGCAUUCGCCCUUGCGUGUCCAUUGUUGCAAGGAGCGAUUUGGAGAAAAUAAACUGUGAGUUU
               ||||||||||||     |            || |||||||||||||||||  |  | | |   |    |||||||||||||
r_ΔmiR(6338)   GGACUUCCAGACCGAACUUCUUUCCGUGUUUUCCGUGUCCAUUGUUGCAAGAUGAAAAUCGAUUAGGGGAAACUGUGAGUUU
               ((((....((((((..........)).)))).....))))..........(((....)))..(((.((....)).)))....
```

---

	miR-BHRF1-1	miR-BHRF1-2	miR-BHRF1-3
AJ507799:	nt 41464-41544	nt 42840-42920	nt 42956-43039
	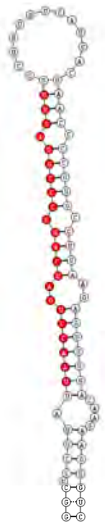	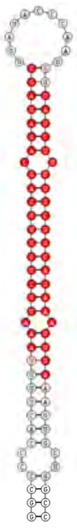	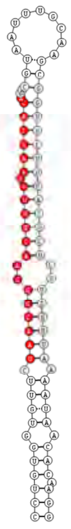
	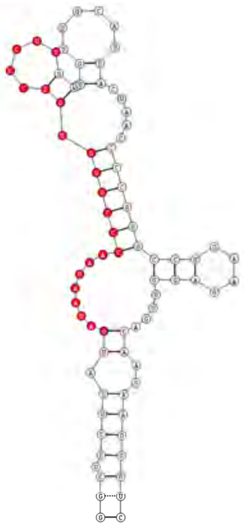	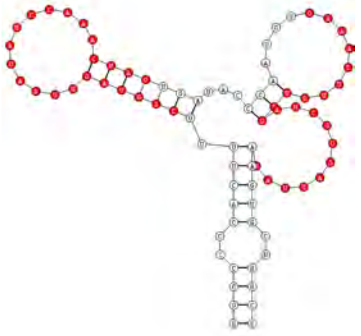	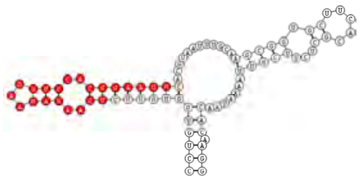
	r_ΔmiR (6338)	r_ΔmiR (6338)	r_ΔmiR (6338)

AJ507799:	miR-BART3<br>nt 139076-139154	miR-BART4<br>nt 139215-139299	miR-BART1<br>nt 139340-139417	miR-BART15<br>nt 139507-139584
	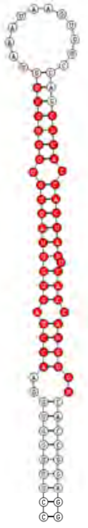	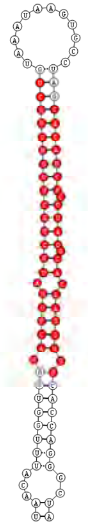	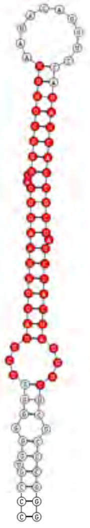	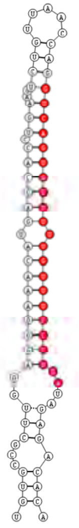
	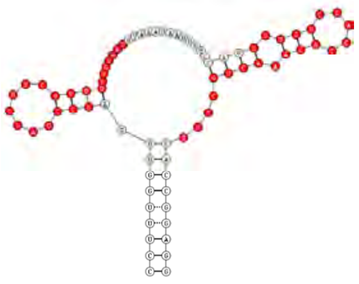<br>r_ΔmiR (6338)	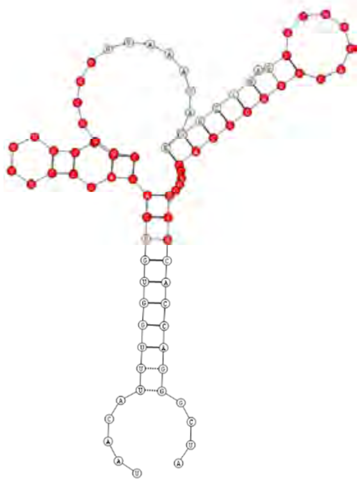<br>r_ΔmiR (6338)	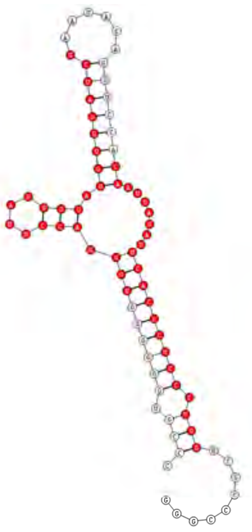<br>r_ΔmiR (6338)	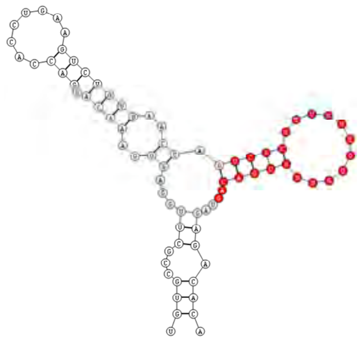<br>r_ΔmiR (6338)

	miR-BART5	miR-BART16	miR-BART17	miR-BART6
AJ507799:	nt 139666-139744	nt 139783-139866	nt 139906-139982	nt 140020-104103
	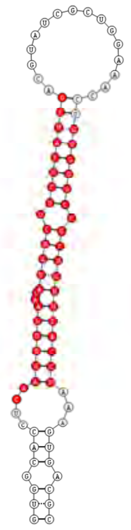	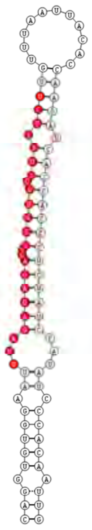	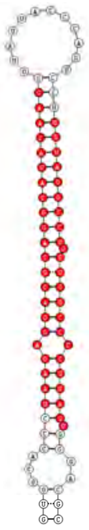	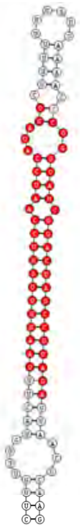
	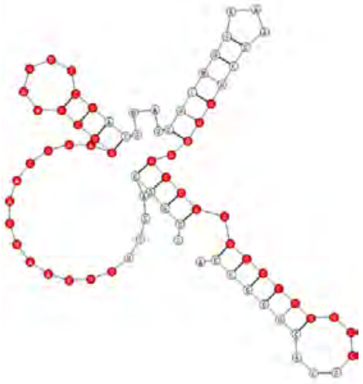	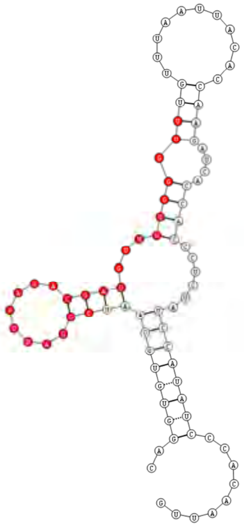	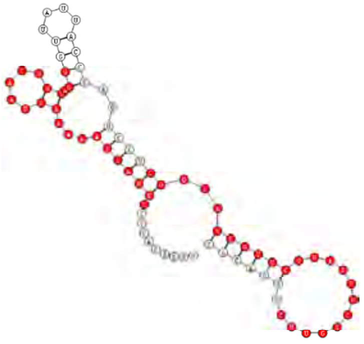	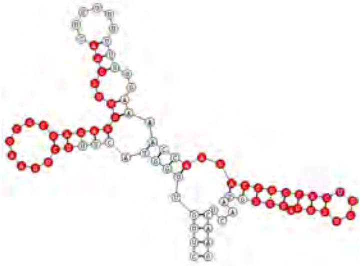
	r_ΔmiR (6338)	r_ΔmiR (6338)	r_ΔmiR (6338)	r_ΔmiR (6338)

	miR-BART21	miR-BART18	miR-BART7	miR-BART8
AJ507799:	nt 145499-145583	nt 145949-145583	nt 146420-146502	nt 146759-146840
	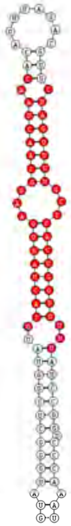	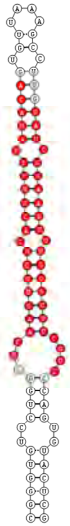	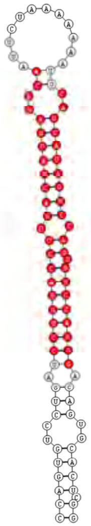	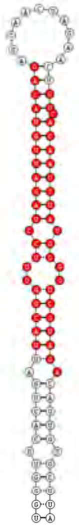
	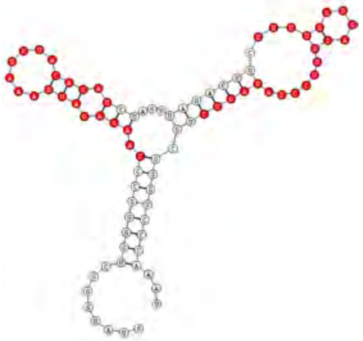 r_ΔmiR (6338)	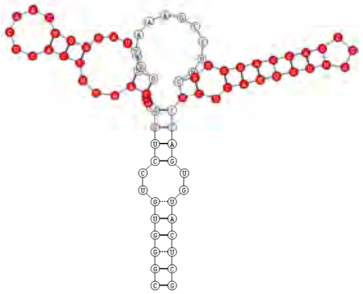 r_ΔmiR (6338)	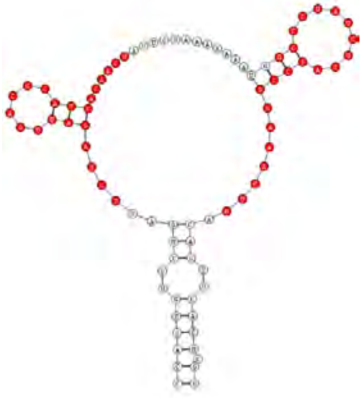 r_ΔmiR (6338)	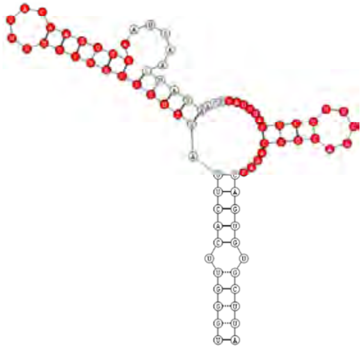 r_ΔmiR (6338)

	miR-BART9	miR-BART22	miR-BART10	miR-BART11
AJ507799:	nt 146947-147031	nt 147155-147236	nt 147308-147388	nt 147524-147609

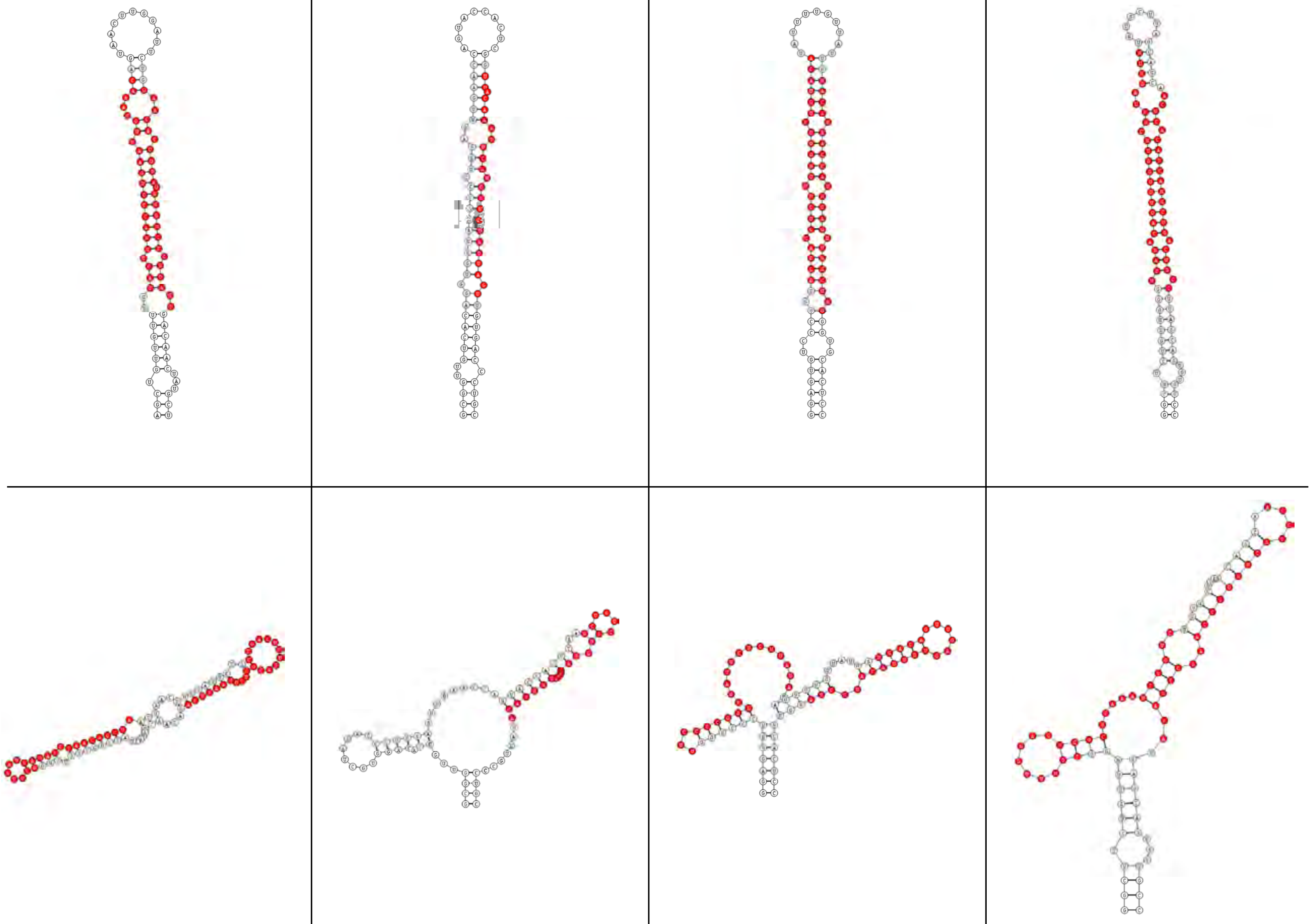

r_ΔmiR (6338)	r_ΔmiR (6338)	r_ΔmiR (6338)	r_ΔmiR (6338)

AJ507799:	miR-BART12<br>nt 147888-147970	miR-BART19<br>nt 148202-148286	miR-BART20<br>nt 148328-148405	miR-BART13<br>nt 148515-148594
	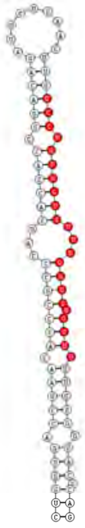	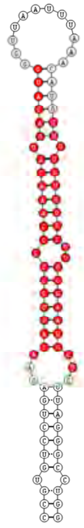	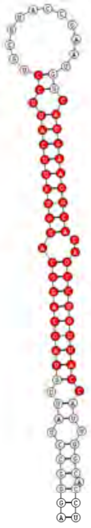	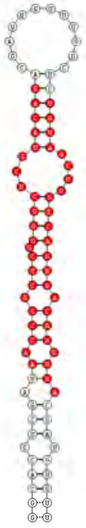
	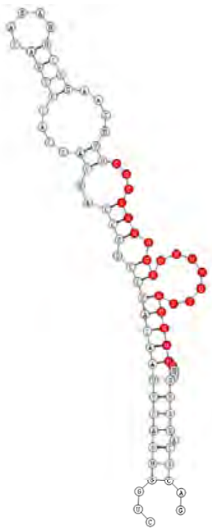<br>r_ΔmiR (6338)	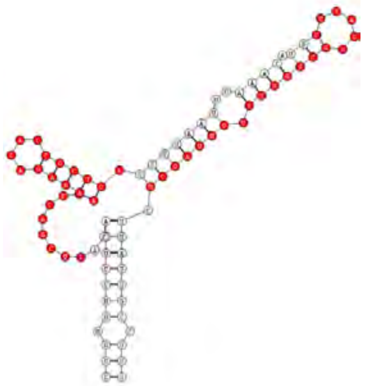<br>r_ΔmiR (6338)	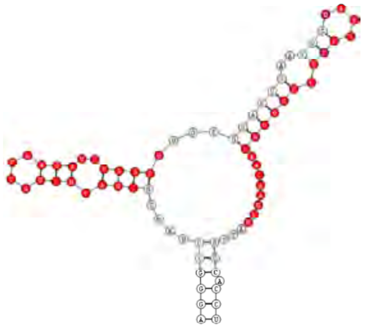<br>r_ΔmiR (6338)	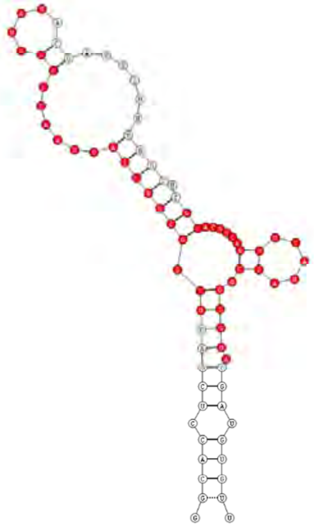<br>r_ΔmiR (6338)

AJ507799:	miR-BART14<br>nt 148731-148815	miR-BART2<br>nt 152735-152816
	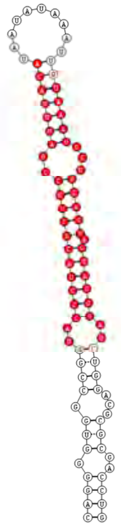	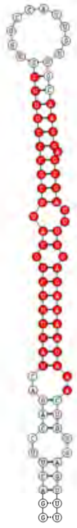
	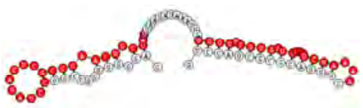<br>r_ΔmiR (6338)	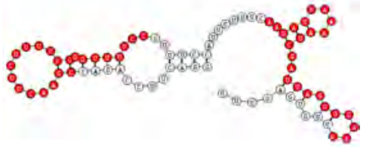<br>r_ΔmiR (6338)
