# Supplementary material for: First Days in the Life of Naive Human B Lymphocytes Infected with Epstein-Barr Virus
Source: mBio. 2019 Sep 17;10(5):e01723-19. doi: 10.1128/mBio.01723-19 (PMC6751056; doi:10.1128/mBio.01723-19)
Supplement: TEXT S1 [file mBio.01723-19-s0001.pdf]

Text S1

DNA sequence of the *rpsL/kana* selection cassette in p6012

```
LOCUS       p6012_rpsL/kana          1348 bp ds-DNA     linear   SYN 29-Oct-2010
DEFINITION           /label=rpsL
                     /gene=rpsL
            MATVNQLVRKPRARKVAKSNVPALEACPQKRGVCTRVYTTTPKKPNSALRKVCRVRLTNGFEVTSYIG
            GEGHNLQEHSVILIRGGRVKDLPGVRYHTVRGALDCSGVKDRKQARSKYGVKRPKA*
                     /label=aph
                     /gene=aph
            MLEQDGLHAGSPAAWVERLFGYDWAQQTIGCSDAAVFRLSAQGRPVLFVKTDLSGALNELQDEAARLS
            WLATTGVPCAAVLDVVTEAGRDWLLLGEVPGQDLLSSHLAPAEKVSIMADAMRRLHTLDPATCPFDHQ
            AKHRIERARTRMEAGLVDQDDLDEEHQGLAPAELFARLKARMPDGEDLVVTHGDACLPNIMVENGRFS
            GFIDCGRLGVADRYQDIALATRDIAEELGGEWADRFLVLYGIAAPDSQRIAFYRLLDEFF*

SOURCE      - synthetic sequence
FEATURES             Location/Qualifiers
     misc_feature    1..24
                     /note="rpsL/kana FW primer"
     CDS             139..513
                     /note="rpsL"
     CDS             554..1348
                     /note="NeoR/KanR"
     misc_feature    complement(1325..1348)
                     /note="rpsL/kana BW primer"
BASE COUNT  277 A  361 C  395 G  315 T  0 OTHER
ORIGIN
        1 ggcctggtga tgatggcggg atcgTTGTAT ATTTCTTGAC ACCTTTTCGG CATCGCCCTA
       61 AAATTCGGCG TCCTCATATT GTGTGAGGAC GTTTTATTAC GTGTTTACGA AGCAAAAGCT
      121 AAAACCAGGA GCTATTTAAT GGCAACAGTT AACCAGCTGG TACGCAAACC ACGTGCTCGC
      181 AAAGTTGCGA AAAGCAACGT GCCTGCGCTG GAAGCATGCC CGCAAAAACG TGGCGTATGT
      241 ACTCGTGTAT ATACTACCAC TCCTAAAAAA CCGAACTCCG CGCTGCGTAA AGTATGCCGT
      301 GTTCGTCTGA CTAACGGTTT CGAAGTGACT TCCTACATCG GTGGTGAAGG TCACAACCTG
      361 CAGGAGCACT CCGTGATCCT GATCCGTGGC GGTCGTGTTA AAGACCTCCC GGGTGTTCGT
      421 TACCACACCG TACGTGGTGC GCTTGACTGC TCCGGCGTTA AAGACCGTAA GCAGGCTCGT
      481 TCCAAGTATG GCGTGAAGCG TCCTAAGGCT TAATGGTAGA TCTGATCAAG AGACAGGATG
      541 ACGGTCGTTT CGCATGCTTG AACAAGATGG ATTGCACGCA GGTTCTCCGG CCGCTTGGGT
      601 GGAGAGGCTA TTCGGCTATG ACTGGGCACA ACAGACAATC GGCTGCTCTG ATGCCGCCGT
      661 GTTCCGGCTG TCAGCGCAGG GGCGCCCGGT TCTTTTTGTC AAGACCGACC TGTCCGGTGC
      721 CCTGAATGAA CTGCAGGACG AGGCAGCGCG GCTATCGTGG CTGGCCACGA CGGGCGTTCC
      781 TTGCGCAGCT GTGCTCGACG TTGTCACTGA AGCGGGAAGG GACTGGCTGC TATTGGGCGA
      841 AGTGCCGGGG CAGGATCTCC TGTCATCTCA CCTTGCTCCT GCCGAGAAAG TATCCATCAT
      901 GGCTGATGCA ATGCGGCGGC TGCATACGCT TGATCCGGCT ACCTGCCCAT TCGACCACCA
      961 AGCGAAACAT CGCATCGAGC GAGCACGTAC TCGGATGGAA GCCGGTCTTG TCGATCAGGA
     1021 TGATCTGGAC GAAGAGCATC AGGGGCTCGC GCCAGCCGAA CTGTTCGCCA GGCTCAAGGC
     1081 GCGCATGCCC GACGGCGAGG ATCTCGTCGT GACCCATGGC GATGCCTGCT TGCCGAATAT
     1141 CATGGTGGAA AATGGCCGCT TTTCTGGATT CATCGACTGT GGCCGGCTGG GTGTGGCGGA
     1201 CCGCTATCAG GACATAGCGT TGGCTACCCG TGATATTGCT GAAGAGCTTG GCGGCGAATG
     1261 GGCTGACCGC TTCCTCGTGC TTTACGGTAT CGCCGCTCCC GATTCGCAGC GCATCGCCTT
     1321 CTATcgcctt cttgacgagt tcttctga
//
```

We constructed a dual selection cassette consisting of the entire E.coli ribosomal S12 gene (*rpsL*) upstream of the aminoglycoside phosphotransferase gene (*aph*), which is also under the control of the *rpsL* promoter. Expression of *rpsL* results in streptomycin sensitivity at 1 mg/ml streptomycin sulfate in *rpsL*-deficient E.coli strains, whereas *aph* expression mediates resistance against 40 µg/ml kanamycin sulfate. This dual selection cassette was cloned onto the pJET1.2 (Fermentas) plasmid to yield the plasmid termed p6012. The entire *rpsL/kana*

selection cassette is 1348 bps in length and can be amplified with a PCR primer pair as specified in the sequence above.

Sequence of the mutation in ΔEBNA1 (6285) compared with its parent

EBNA1 exon in wt/B95.8 (6008)

```
     107950       107960       107970        107980       107990       108000
  TGTGAATC ATG TCT GAC GAG GGG CCA GGT ACA GGA CCT GGA AAT GGC CTA GGA GAG AAG GGA GAC>
  ACACTTAG TAC AGA CTG CTC CCC GGT CCA TGT CCT GGA CCT TTA CCG GAT CCT CTC TTC CCT CTG>
            M   S   D   E   G   P   G   T   G   P   G   N   G   L   G   E   K   G   D >
            ..........BKRF1 encodes EBNA-1 protein...................................
```

EBNA1 exon in ΔEBNA1 (6285)

```
     107950       107960       107970        107980       107990       108000
  TGTGAATC tag TCT GAC GAG GGG CCA GGT ACA GGA CCT GGA AAT GGC CTA GGA GAG AAG GGA GAC>
  ACACTTAG atc AGA CTG CTC CCC GGT CCA TGT CCT GGA CCT TTA CCG GAT CCT CTC TTC CCT CTG>
            *   S   D   E   G   P   G   T   G   P   G   N   G   L   G   E   K   G   D >
           ...........BKRF1 encodes EBNA-1 protein...................................
```

Shown is the start of the EBNA1 encoding exon. In ΔEBNA1 (6285) the point mutations exchanging the start codon of EBNA1 with a stop codon are highlighted. The numerals above the sequence indicate the nucleotide coordinates, which are identical in both EBV genomes.

Sequence of the mutation in ΔEBNA2 (5968) compared with its parent

EBNA2 exon in wt/B95.8 (2089)

```
   48490        48500         48510        48520        48530         48540
  GCT TTA TCT GCC GCC ATC ATG CCT ACA TTC TAT CTT GCG TTA CAT GGG GGA CAA ACA TAT>
  CGA AAT AGA CGG CGG TAG TAC GGA TGT AAG ATA GAA CGC AAT GTA CCC CCT GTT TGT ATA>
                           M   P   T   F   Y   L   A   L   H   G   G   Q   T   Y >
                          ..........BYRF1 encodes EBNA-2 protein.................
```

EBNA2 exon in ΔEBNA2 (5968)

```
   48490        48500         48510        48520        48530         48540
  GCT TTA TCT GCC GCC ATC tga CCT ACA TTC TAT CTT GCG TTA CAT GGG GGA CAA ACA TAT>
  CGA AAT AGA CGG CGG TAG act GGA TGT AAG ATA GAA CGC AAT GTA CCC CCT GTT TGT ATA>
                           *   P   T   F   Y   L   A   L   H   G   G   Q   T   Y >
                          ..........BYRF1 encodes EBNA-2 protein.................
```

Shown is the start of the EBNA2 coding exon; the point mutations replacing the start codon of EBNA2 with a stop codon are highlighted in ΔEBNA2 (5968). The numerals above the sequence indicate the nucleotide coordinates, which are identical in both EBV genomes.

Flanking sequences of the *rpsL/kana* cassette in the EBNA3A locus in ΔEBNA3A (6077) and ΔEBNA3A/C (6331) compared with their parental EBV genomes

EBNA3A in wt/B95.8 (6001) and ΔEBNA3C (6123)

```
       92240          92250          92260           92270                      92670          92680          92690
 TGTTGCAGACAAA ATG GAC AAG GAC AGG CCG GGT CCC CCG GCC>--------<TGGTTTCAG CGC ATC GAC ACA CGA GCC ATA>
 ACAACGTCTGTTT TAC CTG TTC CTG TCC GGC CCA GGG GGC CGG>--------<ACCAAAGTC GCG TAG CTG TGT GCT CGG TAT>
                M   D   K   D   R   P   G   P   P   A >                     <R   I   D   T   R   A   I >
                BLRF3 (spliced to BERF1 to make EBNA3a)                      ...........BERF1..........

              93590          93600          93610          93620
-----<AGG CCG CCT GTT CCG AAA CCA AGA CCA GAG GTC CCA CAA>
-----<TCC GGC GGA CAA GGC TTT GGT TCT GGT CTC CAG GGT GTT>
      <R   P   P   V   P   K   P   R   P   E   V   P   Q >
...........................BERF1 cont. ..................
```

rpsL/kana in ΔEBNA3A (6077) and ΔEBNA3A/C (6331)

```
       92240          92250          92260           92270                      92670          92680          92690
 TGTTGCAGACAAA -----------------------------------rpsL/kana--------------------------------------->
 ACAACGTCTGTTT ----------------------------------------------------------------------------------->

             93590          93600          93610          93620
<---------------- GTT CCG AAA CCA AGA CCA GAG GTC CCA CAA>
<---------------- CAA GGC TTT GGT TCT GGT CTC CAG GGT GTT>
                   V   P   K   P   R   P   E   V   P   Q >
...........................BERF1 cont. ..................
```

In ΔEBNA3A (6077) and ΔEBNA3A/C (6331) the *rspL/kana* selection cassette is used as an insertional mutagen. Shown is the start of the exon with the 5' end of the BLRF3 encoding sequence in wt/B95.8 (6001) or ΔEBNA3C (6123) and the start of the next BERF1 exon with a few nucleotides of the intron in between BLRF3 and BERF1. Gaps which indicate stretches of missing nucleotides are indicated by hyphens (-). The *rspL/kana* selection cassettes in ΔEBNA3A (6077) and ΔEBNA3A/C (6331) were positioned such that they replaced the entire coding sequences of BLRF3 and the 5' part of BERF1. The numerals above the sequences indicate the nucleotide coordinates in wt/B95.8 (6001) and ΔEBNA3C (6123). In ΔEBNA3A (6077) and ΔEBNA3A/C (6331) the nucleotide coordinates downstream of the introduced *rpsL/kana* cassette are not adapted for simplicity. The position of the *rpsL/kana* cassette is shown schematically, only, and indicated in yellow.

Sequence of the mutation in ΔEBNA3C (6123) compared with its parent

EBNA3C in wt/B95.8 (6001)

```
          98750       98760       98770     98780     98790     98800      98810
 <CCT CTA ACT GGG TTC ATG GGG GCC ATC TAAGGCCCACGTGTGACCCATGTTTCCATTAATTTTAG CAA TCG CAC CTG CAA>---
 <GGA GAT TGA CCC AAG TAC CCC CGG TAG ATTCCGGGTGCACACTGGGTACAAAGGTAATTAAAATC GTT AGC GTG GAC GTT>---
   P   L   T   G   F   M   G   A   I>                                       <Q   S   H   L   Q >
.......BERF3 (spliced to BERF4)......                                       .......BERF4.......>
```

```
              98980        98990         99000        99010
----<CCC AGC CAA TCC TGG CCC ATG GGA TAT CGT ACA GCA ACA CTA>
----<GGG TCG GTT AGG ACC GGG TAC CCT ATA GCA TGT CGT TGT GAT>
      P   S   Q   S   W   P   M   G   Y   R   T   A   T   L >
......................BERF4 cont. ..........................
```

EBNA3C in ΔEBNA3C (6123)

```
          98750       98760       98770     98780     98790     98800      98810
 <CCT CTA ACT GGG TTC tga GGG GCC ATC TAAGGCCCACGTGTGACCCATGTTTCCATTAATTTTAG CAA TCG CAC CTG CAA>---
 <GGA GAT TGA CCC AAG act CCC CGG TAG ATTCCGGGTGCACACTGGGTACAAAGGTAATTAAAATC GTT AGC GTG GAC GTT>---
   P   L   T   G   F   *   G   A   I>                                       <Q   S   H   L   Q >
.......BERF3 (spliced to BERF4)......                                       .......BERF4.......>
```

```
              98980        98990         99000        99010
----<CCC AGC CAA TCC TGG CCC tga GGA TAT CGT ACA GCA ACA CTA>
----<GGG TCG GTT AGG ACC GGG act CCT ATA GCA TGT CGT TGT GAT>
      P   S   Q   S   W   P   *   G   Y   R   T   A   T   L >
......................BERF4 cont. ..........................
```

Shown is the end of the BERF3 encoding exon and the start of the BERF4 encoding exon together with the very short intervening intron. The point mutations replacing the two AUG codons with stop codons in the two exons of EBNA3C are highlighted in ΔEBNA3C (6123). The numerals above the sequence indicate the nucleotide coordinates, which are identical in both EBV genomes.

Flanking sequences of the *rpsL/kana* cassettes in ΔEBER (6431) and ΔEBER/ΔmiR (6432) compared with their parents

EBER1 and EBER2 genes in wt/B95.8 (6008) and r_ ΔmiR (6338)

```
       6610      6620      6630      6640      6650      6660      6670
ATGTAGACTTGTAGACACTGCAAAACCTCAGGACCTACGCTGCCCTAGAGGTTTTGCTAGGGAGGAGACGT>-----------
TACATCTGAACATCTGTGACGTTTTGGAGTCCTGGATGCGACGGGATCTCCAAAACGATCCCTCCTCTGCA>-----------
                             |.................EBER 1..................>
```

```
                7110      7120      7130      7140      7150
--------<GAAGGGTATTCGGCTTGTCCGCTATTTTTTTGTGGCTAGTTTTGCACCCAC
--------<CTTCCCATAAGCCGAACAGGCGATAAAAAAACACCGATCAAAACGTGGGTG
        <.............EBER 2.........|
```

rpsl/kana replacing the EBER genes in ΔEBER (6431) and ΔEBER/ΔmiR (6432)

```
       6610      6620      6630      6640      6650      6660      6670
ATGTAGACTTGTAGACACTGCAAAACCTC-----------------------------rpsL/kana----------------->
TACATCTGAACATCTGTGACGTTTTGGAG-------------------------------------------------------->
                             |.................EBER 1..................>
```

```
                7110      7120      7130      7140      7150
<--------------------rpsL/kana-------TTGTGGCTAGTTTTGCACCCAC
<------------------------------------AACACCGATCAAAACGTGGGTG
        <.............EBER 2.........|
```

In ΔEBER (6431) and ΔEBER/ΔmiR (6432) the *rspL/kana* selection cassette is used as an insertional mutagen replacing both EBER1 and EBER2. Shown is the start of EBER1 and the end of EBER2 in wt/B95.8 (6001) and r_ ΔmiR (6338). The *rspL/kana* selection cassettes in ΔEBER (6431) and ΔEBER/ΔmiR (6432) were positioned such that they replaced both EBER loci. The numerals above the sequences indicate the nucleotide coordinates in wt/B95.8 (6001) or r_ ΔmiR (6338) and are not corrected in ΔEBER (6431) or ΔEBER/ΔmiR (6432) downstream of the introduced *rpsL/kana* cassettes for simplicity. The position of the *rpsL/kana* cassette is shown schematically, only, and indicated in yellow.
